# The Songbird Neurogenomics (SoNG) Initiative: Community-based tools and strategies for study of brain gene function and evolution

**DOI:** 10.1186/1471-2164-9-131

**Published:** 2008-03-18

**Authors:** Kirstin Replogle, Arthur P Arnold, Gregory F Ball, Mark Band, Staffan Bensch, Eliot A Brenowitz, Shu Dong, Jenny Drnevich, Margaret Ferris, Julia M George, George Gong, Dennis Hasselquist, Alvaro G Hernandez, Ryan Kim, Harris A Lewin, Lei Liu, Peter V Lovell, Claudio V Mello, Sara Naurin, Sandra Rodriguez-Zas, Jyothi Thimmapuram, Juli Wade, David F Clayton

**Affiliations:** 1 Cell & Developmental Biology, Univ. of Illinois, Urbana, IL, USA; 2 Physiological Sci., UCLA, Los Angeles, CA, USA; 3 Psychological & Brain Sci., Johns Hopkins Univ., Baltimore, MD, USA; 4 Psychology, Biology, and Bloedel Hearing Research Center, Univ. of Washington, Seattle, WA, USA; 5 Mol. & Integrative Physiology, Univ. of Illinois, Urbana, IL, USA; 6 Neurological Sci. Inst., Oregon Hlth. Sci. Univ., Beaverton, OR, USA; 7 Psychology, Zoology & Neuroscience, Michigan State Univ., East Lansing, MI, USA; 8 W.M. Keck Center for Comparative & Functional Genomics, Univ. of Illinois, Urbana, IL, USA; 9 Institute for Genomic Biology, Univ. of Illinois, Urbana, IL, USA; 10 Animal Sciences, Univ. of Illinois, Urbana, IL, USA; 11 Animal Ecology, Lund University, S-223 62 Lund, Sweden; 12 Neuroscience Program, Univ. of Illinois, Urbana, IL, USA

## Abstract

**Background:**

Songbirds hold great promise for biomedical, environmental and evolutionary research. A complete draft sequence of the zebra finch genome is imminent, yet a need remains for application of genomic resources within a research community traditionally focused on ethology and neurobiological methods. In response, we developed a core set of genomic tools and a novel collaborative strategy to probe gene expression in diverse songbird species and natural contexts.

**Results:**

We end-sequenced cDNAs from zebra finch brain and incorporated additional sequences from community sources into a database of 86,784 high quality reads. These assembled into 31,658 non-redundant contigs and singletons, which we annotated via BLAST search of chicken and human databases. The results are publicly available in the ESTIMA:Songbird database. We produced a spotted cDNA microarray with 20,160 addresses representing 17,214 non-redundant products of an estimated 11,500–15,000 genes, validating it by analysis of immediate-early gene *(zenk) *gene activation following song exposure and by demonstrating effective cross hybridization to genomic DNAs of other songbird species in the Passerida Parvorder. Our assembly was also used in the design of the "Lund-zfa" Affymetrix array representing ~22,000 non-redundant sequences. When the two arrays were hybridized to cDNAs from the same set of male and female zebra finch brain samples, both arrays detected a common set of regulated transcripts with a Pearson correlation coefficient of 0.895. To stimulate use of these resources by the songbird research community and to maintain consistent technical standards, we devised a "Community Collaboration" mechanism whereby individual birdsong researchers develop experiments and provide tissues, but a single individual in the community is responsible for all RNA extractions, labelling and microarray hybridizations.

**Conclusion:**

Immediately, these results set the foundation for a coordinated set of 25 planned experiments by 16 research groups probing fundamental links between genome, brain, evolution and behavior in songbirds. Energetic application of genomic resources to research using songbirds should help illuminate how complex neural and behavioral traits emerge and evolve.

## Background

Songbirds offer unique opportunities for studying the links between genome, brain structure, neurophysiology, behavior and evolution. They are one of the most successful vertebrate radiations, diversifying into more than 4000 different species over the past ~65 million years [1,2] and displaying a great range of physical, behavioral and social adaptations [3,4]. Many songbird species are readily observed in their natural habitats and several species have been domesticated (e.g., zebra finch, canary). All songbirds share a highly specialized neural system for learned vocal communication. Indeed, songbirds are one of the few animal groups capable of complex vocal learning and stand alone as accessible experimental models relevant to human speech [5,6]. Neurobiological research using songbirds has consistently generated new insights that were later found to be true for other vertebrates; examples include definitive evidence for sexual differentiation of brain circuits, seasonal changes in brain anatomy, and lifelong neuronal replacement [7].

Against these opportunities, the songbird as a research model also presents challenges. Although the zebra finch has emerged as a primary focus, many other songbird species are studied especially in natural contexts. It will be important to develop tools and reagents that allow study not just of one species (the zebra finch) but of many, so as to exploit the diversity of vocal behavior shown by songbirds. Some of these studies will be comparative in nature (e.g., how is the genome different in species with lifelong vocal learning, compared to species that learn song only once?). Other research objectives may focus on a single wild-caught species to address questions of gene regulation and variation in a particular niche, environment or behavioral paradigm. A related challenge is that researchers with expertise in field biology, neuroscience, physiology or behavioral analyses may not necessarily have equal expertise in molecular genetics, and vice versa.

In response to these needs and opportunities, in 2002 we inaugurated the Songbird Neurogenomics (SoNG) Initiative [8,9]. Our goals were to leverage rapid advances in genomic technology, so as to bring new resources to songbird research and stimulate collaborative approaches that would integrate field researchers, physiologists and molecular biologists. Here we describe the following accomplishments, which form the foundations of the ongoing SoNG Initiative: 1) three generations in the development of an evolving catalog of gene sequences expressed in the zebra finch brain; 2) production and validation of DNA microarrays useful for study of diverse songbird species; 3) organization of an efficient community-based mechanism for stimulating use of these resources.

## Results

### High-Throughput Expressed Sequence Analysis and Annotation

Over the course of five years, we generated three sequential assemblies of expressed sequence information, each one incorporating more data drawn from both our own primary sequencing efforts and from other zebra finch research groups working in parallel (Table 1). For efficient generation, annotation and presentation of expressed sequence information, we made use of the ESTIMA software interface [10] and a production pipeline that had been refined in development of resources for cattle [11], honeybees [12] and other emerging research organisms [13]. In the first phase of the project, we applied high-throughput DNA sequencing methodologies to generate a collection of Expressed Sequence Tags (ESTs) from a normalized zebra finch brain cDNA library. The resulting sequence information was assembled (ESTIMA:Songbird Build 1), made publicly available via the internet [9] in 2004, and used to build the cDNA array described later in this report. In the second phase of the project, we incorporated sequence information obtained from a sister project [14]. The sequences were re-assembled (ESTIMA:Songbird Build 2, replacing Build 1 on the SoNG Initiative website in 2005) and used in fabrication of an Affymetrix array as described [15]. In the third phase, we incorporated additional data from subtractive sequencing of our original cDNA library and data from another sister project [16] (ESTIMA Build 3, replacing Build 2 in October 2007).

**Table 1:** Sequence sources and statistics for the three sequential ESTIMA:Songbird assemblies

			**ESTIMA:Songbird**		
			**BUILD 1**	**BUILD 2**	**BUILD 3**
			
*Sequence Source*	*Raw reads*	*After filtering*	*20 K cDNA microarray*	*Lund/Affy microarray*	
SB01	2,880	1,840	√	√	√
SB02	18,720	16,360	√	√	√
SB03 (a)	19,104	17,032	√	√	√
SB03 (b)	9,120	5,717		√	√
Duke chromatograms	36,469	23,225		√	
Duke from NCBI	17,312	16,442			√
SB05	11,328	10,056			√
SB06	11,040	9,760			√
Rockefeller	9,845	9,764			√

*Raw reads*			*40,704*	*86,293*	*99,349*
**# SEQS INTO PTA**			**35,232**	**58,457**	**86,784**
*Pure singlets*			*10,123*	*11,823*	*13,908*
*Clustered singlets*			*1,900*	*2,457*	*3,662*
*Total singlets*			*12,023*	*14,280*	*17,570*
C*ontigs*			*5,855*	*8,348*	*14,088*
**Total unique sequences**			**17,878**	**22,628**	**31,658**
Total redundant sequences			*17,354*	*35,829*	*55,126*
Redundancy			*49%*	*61%*	*64%*

#### ESTIMA:Songbird Build 1

For the primary EST assembly used in production of the SoNG 20 K array (below), cDNA clones were 5'-end-sequenced using high-throughput methods. 35,232 filtered high-quality ESTs were clustered and assembled into 17,878 non-redundant sequences (Table 1), comprising 10,120 pure singlets, 5,855 contigs and an additional 1,903 clustered singlets (i.e., some sequence relationships in initial cluster analysis but could not be assembled into unbroken contigs; possible splice variants). In subsequent EST builds and BLAST analyses (below), 2900 of the Build 1 sequences were absorbed into larger contigs or aligned against redundant gene targets in other species, and 3409 failed to align anywhere (including the whole genome assembly of the chicken). Thus we estimate Build 1 and the corresponding SoNG 20 K microarray represent products coming from between 11,569 and 14,978 unique genes, with the uncertainty depending on the origin of the 3409 ESTs that cannot yet be aligned with a known orthologous genomic region.

#### ESTIMA:Songbird Build 2

The ESTIMA:Songbird database was designed to be a dynamic repository for zebra finch cDNA sequences in an evolving collection. A complementary initiative centered at Duke University emphasized the isolation of full-length cDNA clones from libraries representing a diversity of zebra finch brain states [14]. The Duke investigators provided us with sequencing chromatograms from their project, and we combined this with our SB01-02-03 sequence data to produce a single assembly representing 58,457 filtered high-quality reads and 22,628 assembled unique sequences (Table 1). This assembly was used as the basis for design of a short-oligo Affymetrix array in a parallel project at Lund University [15] (see below). To estimate gene representation empirically, we performed a test BLAST search for 61 orthologs of specific interest across a range of functional categories (Table 2). Forty-one (~67%) were present in Build 2, including representatives of each of the functional categories of interest. However, numerous gaps in coverage remain, as shown for example by absence of several important steroid receptors and channel proteins in the assembly.

**Table 2:** ESTIMA:Songbird Build 2, presence ("yes") or absence ("no") of specific sequences of interest assessed by direct BLAST with available avian or mammalian sequences against 58,211 sequences in the ESTIMA database as of June 2005.

	*Yes*	*No*		*Yes*	*No*		*Yes*	*No*
**Steroid Receptors**			**Extracellular Signaling**			**GABA transmission**		

Androgen Receptor		•	BDNF	√		GABA-A alpha 1	√	
estrogen receptor alpha		•	cannabinoid receptor		•	GABA-A alpha 2	√	
estrogen receptor beta		•	IGF-2	√		GABA-A alpha 4		•
estrogen receptor gamma	√		N-CAM	√		GABA-A alpha 5	√	
RARalpha	√					GABA-A beta 3	√	
RARbeta	√		**Intracellular signaling**			GABA-A delta	√	
RARgamma		•	Calbindin	√		GABA-A gamma 2	√	
RXRalpha	√		CAMKII	√		GABA-A gamma 3/4	√	
RXRbeta		•	Canarygranin	√		GABA-B	√	
RXRgamma		•	CRABPI	√		GABA-C rho-2B		•
**Steroid Metabolism**			CRABPII	√		GAD-65	√	
Aromatase	√		CRBPI		•	GAD67	√	
P450-CYP26		•	CRBPII		•	**Potassium channels**		
zRalDH	√		GAP-43	√		Kcnma1		•
**Immediate Early Genes**			MAPKK	√		Kcnmb1		•
ARC	√		Narp	√		Kcnmb2		•
c-fos	√		parvalbumin	√		Kcnmb3		•
c-jun	√		PAX-6	√		Kcnmb4		•
*zenk*	√		SNAP-25	√		Kv3.1	√	
**Cytoskeleton**			Synapsin I		•	**Transporters**		
Beta Tubulin	√		synapsin IIb	√		VGAT		•
GFAP	√					VGLUT		•
NFM	√					VIAAT	√	
vimentin	√							

#### ESTIMA:Songbird Build 3

In a third build of the database, we incorporated two more rounds of subtractive sequencing plus additional sequences from colleagues at Rockefeller University [16]. The combined total of high-quality filtered reads now reached a total of 86,784, which assembled into 31,658 non-redundant transcripts (Table 1). Having reached a marginal redundancy of 64%, we suspended further sequencing of this cDNA source. However, the frequency of singletons remains high (~55%) indicating considerable sequence diversity still remains unrepresented.

#### Annotation

We performed machine annotations of each of the sequential ESTIMA builds by BLAST sequence similarity searches against external databases, making the results publicly available through the ESTIMA:Songbird website [9]. For Build 3, the BLAST analyses aligned 77% of the assembled unique sequences to putative orthologs in other vertebrates (Table 3A). For nomenclature, we used the International Protein Index (IPI) of the European Bioinformatics Institute (EBI) as a primary reference point [17]. IPI is a database of cross references between the primary data sources, providing a minimally redundant yet maximally complete set of protein sequences and stable identifiers for featured species (one sequence per transcript). IPI also maps to a growing set of qualified Gene Ontology (GO) terms for its sequence entries. Searching against chicken IPI, we obtained alignments for 13,219 (42%) of our assembled Build 3 sequences (Table 3B).

**Table 3:** Annotation statistics for ESTIMA:Songbird Build 3

	**Build 3**	*Percent total*	**20 K array subset**	*Percent of array*	*Percent of Build 3 category*
*Unique sequences after assembly*	***31658***	*100%*	***17214***	*100%*	*54%*
**A) ALL HITS BY DATABASE**					
Gga Genome	21601	*68%*	12396	*72%*	*57%*
Chicken_TC	20208	*64%*	11715	*68%*	*58%*
Gga Unigene	17980	*57%*	10490	*61%*	*58%*
NCBI_Chicken_RNA	15904	*50%*	9110	*53%*	*57%*
Ensembl_Chicken_cdna_all	14609	*46%*	8348	*48%*	*57%*
Ensembl_Chicken_cdna_abinitio	13223	*42%*	7588	*44%*	*57%*
Chicken IPI	13219	*42%*	7898	*46%*	*60%*
Hs Unigene	12373	*39%*	7224	*42%*	*58%*
NCBI_Chicken_protein	7776	*25%*	4517	*26%*	*58%*
	
*Hits against any database*	***24466***	*77%*	***13803***	*80%*	*56%*

**B) Hierarchy of CUSTOM ANNOTATION**					
1 Use IPI-annotation*	13219	*42%*	7553	*44%*	*57%*
2 in GGA_Unigene but not IPI	5614	*18%*	3364	*20%*	*60%*
3 in HS but not IPI or GGA_unigene	265	*1%*	104	*1%*	*39%*
	
*Total number of "custom annotations"*	*19098*	*60%*	11021	*64%*	*58%*
4 additional "conserved in chicken"	5368	*17%*	2784	*16%*	*52%*
	
*Hits against any database*	*24466*	*77%*	13805	*80%*	*56%*
5 remainder = "TGU-specific" (no hits)	7192	*23%*	3409	*20%*	*47%*
	
	31658	*100%*	17214	*100%*	*54%*

*C) IPI Annotations					
Number of sequences with IPI identifiers	13219		7898	(Unigene used for 345)	
Number of unique IPI identifiers	**8127**		**6035**		
			
*All identifiers in IPI release 3.26*	*25500*		*25500*		
Fraction of total IPI identifiers	32%		24%		

In addition to the 13,219 sequences that aligned with IPI records, another 5614 found matches against the chicken Unigene database but not IPI, and a further 265 aligned against Human Unigene but neither Chicken Unigene nor IPI (Table 3C). In sum, we were able to assign "custom annotation" names for 19,098 sequences, or 60% of the Build 3 assembly. A further 17% showed significant alignment to the chicken genome at sites with no current functional gene annotation, and the remaining 23% have not yet been aligned to any putative orthologous genomic region.

### Microarray Production and Validation

For gene expression studies, two different DNA microarrays were produced from the first two ESTIMA:Songbird builds (Table 1). After ESTIMA:Songbird Build 1, the sequenced cDNA inserts were PCR-amplified and spotted onto a glass microarray; for contigs, a single representative clone was spotted (Methods). In addition to these clones, we also added 36 previously characterized cDNA clones as controls on the array (Tables 4 and 5). The total number of addresses on each array = 20,215 (hence we refer to this as the **SoNG 20 K** array). An annotation file for this array based on the Songbird3 Assembly is presented as Additional File 1. From ESTIMA:Songbird Build 2, a separate short oligonucleotide array (**Lund-zfa** array) was produced using sets of 25 mer "perfect match" probes designed by Affymetrix [15] (additional information about this array is provided in Methods). We performed three detailed analyses to assess the performance of these microarrays to validate their use for specific research objectives.

**Table 4:** Content of SoNG 20 K array

Unique sequences in SB1 assembly	17878
*Unique in SB3 assembly*	*17214*
*Redundant in SB3 contigs*	*664*
Controls and knowns	368
Spots that replicate SB clones	967
Total DNA spots on array	19213
Blanks and buffer spots	950

**Total addresses on array**	**20160**

**Table 5:** Previously Cloned Genes and Replicated Controls spotted on 20 K Array

**Seq ID (Gal file)**	**Provided by**	**Genbank Accession**	**Notes**
AR	Art Arnold		Androgen receptor
ARC	Claudio Mello		
beta actin	SB02028A1F09.f1	CK311416	Replicated control
CAB	Keck^1^	BE190670	Replicated control (soybean)
ER alpha	Art Arnold		Estrogen Receptor alpha
ER beta	Art Arnold		Estrogen Recetor beta
GAPDH	SB02023A1H08.f1	CK304281	Replicated control
Histone H3	SB02008B2G06.f1	CK315888	Replicated control
KA1	K. Wada	AB107130	Wada et al.^2^
KA2	K. Wada	AB107131	Wada et al.^2^
MSG	Keck^1^	AJ239127	Replicated control (soybean)
RAR alpha	Claudio Mello	AY714582	Retinoic Acid Receptor
RAR beta	Claudio Mello	AY714583	Retinoic Acid Receptor
RAR gamma	Claudio Mello	AY714584	Retinoic Acid Receptor
RUB	Keck^1^	AI495218	Replicated control (soybean)
*zenk*	David Clayton	EF050732	Replicated control (canary "e12" clone)
zf GluR1	K. Wada^2^	AB042749	AMPA subunit, Wada et al.^2^
zf GluR2	K. Wada^2^	AB042750	Wada et al.^2^
zf GluR3	K. Wada^2^	AB042751	Wada et al.^2^
zf GluR4	K. Wada^2^	AB042752	Wada et al.^2^
zf GluR5	K. Wada^2^	AB107127	Kainate, Wada et al.^2^
zf GluR6	K. Wada^2^	AB107128	Wada et al.^2^
zf GluR7	K. Wada^2^	AB107129	Wada et al.^2^
zf mGluR1	K. Wada^2^	AB042753	Metabotropic, Wada et al.^2^
zf mGluR2	K. Wada^2^	AB042754	Wada et al.^2^
zf mGluR3	K. Wada^2^	AB107132	Wada et al.^2^
zf mGluR4	K. Wada^2^	AB042755	Wada et al.^2^
zf mGluR5	K. Wada^2^	AB107133	Wada et al.^2^
zf mGluR8	K. Wada^2^	AB107134	Wada et al.^2^
zf NR1	K. Wada^2^	AB042756	NMDA, Wada et al.^2^
zf NR2A	K. Wada^2^	AB042757	Wada et al.^2^
zf NR2B	K. Wada^2^	AB107125	Wada et al.^2^
zf NR2C	K. Wada^2^	AB042758	Wada et al.^2^
zf NR2D	K. Wada^2^	AB042759	Wada et al.^2^
zf NR3A	K. Wada^2^	AB107126	Wada et al.^2^
zf *ZENK*	Claudio Mello		*ZENK *cDNA clone ZZF23

^1^contributed to Keck Center by Lila Vodkin ^2^Wada K et al, JCN 476:44-64 (2004)

#### ZENK Hybridization Analysis (SoNG 20 K array)

**Figure 1:**
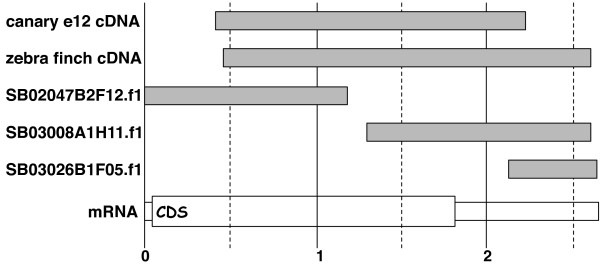
**Schematic alignment of *zenk *sequences on array**. Five partial *zenk *cDNAs are spotted on the 20 K microarray. Here they are aligned against the derived contig ("mRNA") and its predicted protein coding sequence ("CDS"). Canary e12 cDNA [Genbank: EF050732] is the original songbird *zenk *sequence [20]. Zebra finch ZZF23 cDNA [Genbank: EF052676] was isolated using the canary sequence to probe a zebra finch cDNA library (C.V. Mello et al., unpublished). The three clones beginning in "SB..." are three independent cDNAs that did not form contigs and were spotted independently on the array; sequence extent is based on raw EST sequence. Scale bar in kb from putative 5' end of mRNA.

One of the most-studied examples of differential gene expression in the songbird brain is the induction of the immediate early gene *zenk *in the auditory lobule (AL) of the telencephalon following presentation of song playbacks [18-28]. We used this phenomenon as the basis for a primary test of the 20 K microarray. The original canary cDNA clone for study of *zenk *expression in both canaries and zebra finches [20] was spotted redundantly on the array as a replicated internal control. Additionally, a near-full-length cDNA from the zebra finch was spotted once on each array as one of the "known" genes. Fortuitously, three other cDNAs representing distinct parts of the transcript were also present on the array, as they did not form contigs in our EST sequence assembly (Fig. 1). Thus the microarray contains 5 different probes spanning the *zenk *mRNA and also representing the sequences of two different songbird species.

For this first experiment we used 6 microarrays and a simple direct-comparison design, where each array was hybridized with two probes from a different pair of zebra finches. For each pair, one bird heard a 30 minute song stimulus and the other bird heard only silence. They were immediately euthanized and total RNA was extracted from the dissected ALs. We analyzed the hybridization data using a two-stage parametric linear modeling approach (Methods). Data for the five *zenk*-hybridizing probes are summarized in Table 6.

**Table 6:** *zenk*-hybridizing sequences on the 20 K microarray (ordered as in Figure 1).

**ID**	**Description**	**Fold Increase**^a^	**Raw p**^b^	**FDR**^c^	**Mean intensity**^d^
Canary e12 cDNA	Replicated control	2.0	1.5E-59	0.00	3661
Zebra finch cDNA	Single-spotted control	2.1	2.1E-03	0.16	3602
SB02047B2F12.f1	5'-most EST	1.2	1.0E-01	0.35	9005
SB03008A1H11.f1	Central EST	1.7	3.6E-04	0.15	5551
SB03026B1F05.f1	3'-most EST	2.3	7.6E-06	0.05	4799

a. Mean of intensity ratios for song-stimulated sample divided by silence control sample (n = 6 pairs).

b. P-value (uncorrected for multiple testing) of null hypothesis that song and silence groups have the same distributions, derived from a 2-stage parametric linear model (Methods).

c. False Discovery Rate [81].

d. Mean of all measurements (all replicate spots against all samples); background intensities were less than 60 in all cases.

Each probe reported an increased signal in the song-stimulated samples versus controls. The redundantly replicated canary *zenk *control probe measured a two-fold increase in hybridization signal in the song-stimulated samples, somewhat smaller than in past studies where signal for this probe was localized in histological sections by in situ hybridization [20,21,29]. Nevertheless the increase was observed here with high statistical confidence (FDR effectively zero). Both the canary and zebra finch control cDNAs reported similar fold-changes and mean signal intensities, suggesting that these hybridization conditions support effective species cross-hybridization for *zenk*. For the other three EST-derived probes, the magnitudes of increase ranged from 1.2-fold to 2.3-fold. Interestingly, the 5'-most probe measured the smallest fold-change but had the largest mean signal intensity, with the converse for the 3'-most probe. These differences may reflect differential rates of RNA processing along the transcript or sequence relationships to other cross-hybridizing molecules (e.g., zinc finger domains).

#### Concordance of SoNG 20 K and Lund-zfa arrays

Comparing microarray analyses between different platforms and labs is a difficult task and the results have to be interpreted cautiously. These problems include not only differences in array design and fabrication but also differences in sampling handling [30-32] and differences in the approaches to analyse data from cDNA and short-oligo Affymetrix arrays. Although we acknowledge these problems and the fact that quantitative comparisons are very difficult, we still believe that it is useful to qualitatively compare our two zebra finch array platforms. To the degree the platforms give fundamentally similar results, they validate each other. To compare the SoNG 20 K array and the Lund-zfa array, a set of 11 samples (5 adult males, 6 adult females) were hybridized to both array types.

On the SoNG 20 K array, between 98.4% and 99.8% of the spots on each array (excluding the negative controls) were detectable above local background by the GenePix software, indicating that almost all of the cDNAs on the array are expressed in each of the male and female adult brains (Methods). On the Lund-zfa array, when we used a global background calculated from the cells with the 2% lowest signals + 2 SD we found that 98.7% – 99.9% of the probes were detectable above background (Methods). Since each EST on an Affymetrix array is represented by 11 probes and only 8 probes need to be detectable for significance, virtually all of the ESTs on the arrays will be detected. Hence, both platforms are similar in this rough measure of sensitivity and both detect almost all the transcripts in male and female adult brains.

To compare detection of differentially expressed RNAs, we focused only on those singletons/contigs that could be mapped in a 1:1 relationship across the two platforms, i.e., represented by one clone on the SoNG 20 K array and one probe set on the Lund-zfa array. This resulted in 15,537 comparable gene targets (hereinafter "genes") on each array type (Methods). At a False Discovery Rate (FDR) of 0.05, 492 genes were significantly different between males and females on the SoNG 20 K array, versus 355 genes on the Lund-zfa array. Note that the quantitative differences between the two significant lists are not directly comparable due to differences in sample handling and array analyses. However, despite this the lists shared 286 genes in common, such that most of the expression differences found on the Lund-zfa arrays were also found on the SoNG 20 K array (80.5%). Hence, the fact that so many of the sexually dimorphic genes were identified by both array types shows that the results from the two platforms are in concordance. Presentation and analysis of sex-regulated genes *per se *is the subject of a separate investigation (A. Arnold and J. Wade, in preparation) to be reported elsewhere.

**Figure 2:**
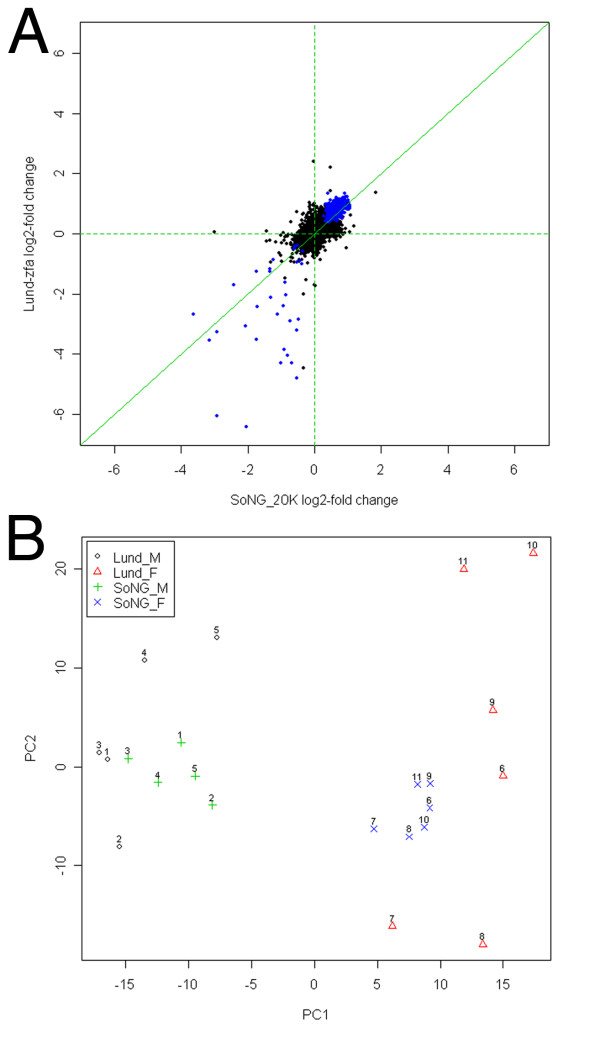
**Comparison of SoNG 20 K cDNA array and Lund-zfa array performance**. A) Correlation plot comparing hybridization data (for 15,537 gene targets) on the Lund-zfa array (vertical axis) versus the SoNG 20 K array (horizontal axis). Each point shows the mean log2 ratio (male:female) for a single gene. Blue indicates significant difference between males and females (FDR = .05) on both arrays. See Results for discussion of correlation coefficients. B) Principal Components Analysis (PCA) of all data on both array platforms. Each data point represents an array, hybridized to the RNA sample indicated by the adjacent number (1–5 are males and 6–11 are females). The key indicates the array platform. For each gene on each array, expression value was scaled to the overall mean for that gene on that platform. Note that the first principal component (PC1), which explains 16.2 % of the variation, reveals a major effect of sex in the data, independent of platform.

Moreover, we also compared the estimated log2-fold changes for all genes on both array types (Figure 2A). The overall Pearson correlation coefficient is 0.573; while this may not seem very high, the separate analyses of each array type indicate that > 95% of the genes likely have zero fold-change plus random measurement error, so they are not expected to be correlated [31]. The correlation coefficient between genes with significant expression differences on both arrays is 0.895. This clearly shows that both platforms to a large degree identify the same biological variation.

We conducted a principle components analysis of all 22 sets of array values (using the 15,537 genes in common) after scaling the pre-processed expression values to the overall mean for each gene (Figure 2B). Interestingly, the first principle component (PC1), which explains 16.2 % of the variation, separates the arrays by sex. This shows that the effect of sex is much more important than any variation between platforms that is measured by the other principal components, and that both array platforms are efficient in picking up the biological variation between the samples. The array difference shows up in PC2 (10.3%) & PC3 (8.5%), while the sample pairs do not segregate together until PC4 & PC5 (7.2% and 6.5%). The larger between-sample variation in PC2 (Figure 2B) for the Lund-zfa array compared to the SoNG 20 K array may reflect technical and analytical differences between the hybridizations on the two platforms (for example sample treatment), and does not necessarily indicate an intrinsic difference in the quality of the two arrays.

#### Comparative genomic hybridization assessment

A major goal of the SoNG Initiative is to facilitate application of genomic analysis to the broad range of songbird species now used in research. To assess the efficacy of our 20 K zebra finch array for measuring gene expression in other songbirds, we performed a set of Comparative Genomic Hybridizations (CGH), where instead of reacting the arrays with labeled tissue mRNA (cDNA), we hybridized with labeled genomic DNA from each of the species. In this way, any differences in signal intensity can be attributed directly to sequence differences between the species, not confounded by differences in gene expression. In general, avian species have a substantially lower repeat density than do mammals, simplifying the analysis of genomic DNA hybridizations [33,34].

For CGH analysis, we obtained DNA from four other species representative of those proposed for study by the SoNG Initiative collaborators (see next sub-section). White-crowned sparrows and song sparrows are common subjects for field research in North America; the canary is a domesticated species typically studied in the laboratory. Like the zebra finch, all are members of the superfamily Passeroidea. The European starling is studied both in the wild and in captivity. As a member of a different superfamily (superfamily Muscicapoidea), the starling is somewhat more distant from the zebra finch. For comparison we also obtained DNA from two even more distantly related birds, the kingbird and the chicken. The kingbird is a suboscine; like songbirds, suboscines are also members of Order Passeriformes, but suboscines have no apparent vocal learning ability and are estimated to have diverged from oscines (songbirds) about 77 MYA [1]. We also obtained DNA from chickens, a member of a different order of birds that diverged from Passeriformes approximately 100 MYA [35]. Each species was represented by four separate individuals, 2 males and 2 females. Each sample from an individual bird was hybridized on a separate array along with a universal reference sample of genomic DNA pooled from 4 zebra finches (two males and two females) labeled with Cy5. The individual samples were labeled with Cy3 and the universal reference with Cy5 (for our general purpose here we considered the impact of dye bias to be negligible). The resulting data were then expressed as the log(2) ratios of the signal in the two dye channels for each spot on the arrays. A log(2) ratio of zero indicates equal signal from the test species and the reference genomic DNAs.

**Figure 3:**
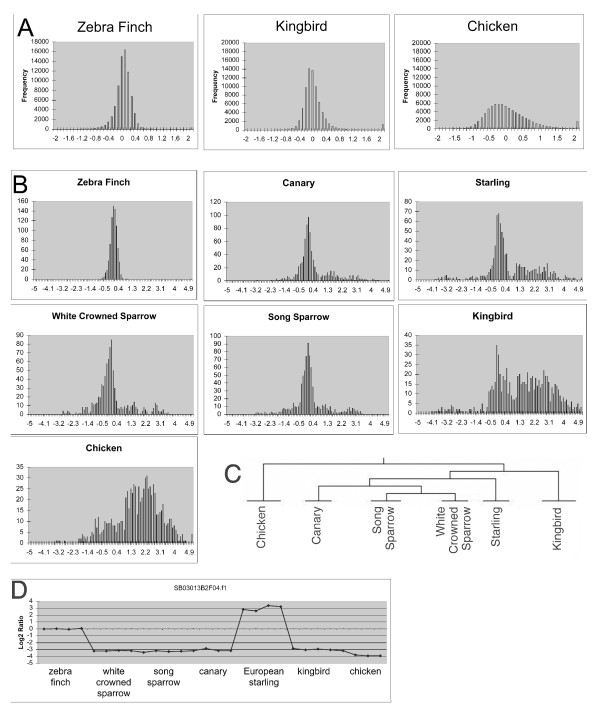
**CGH analyses**. A) For each species, genomic DNAs from each animal (n = 4) were hybridized on separate arrays against a reference DNA from pooled zebra finches. Histograms are shown of the resulting mean log2 ratios (zebra finch:test species) for all gene targets, by test species. Note that when samples from individual zebra finches were hybridized versus the pooled zebra finch reference, the log2 signal ratios cluster closely around 0 as expected. The curve becomes shorter and broader with cross-hybridization to the kingbird and the chicken. B) Histograms of log2 ratios (zebra finch:test species) for a subset of 200 spots found significantly different on multi-species analysis of CGH data. Significant spots were determined by multi-class SAM Analysis using TIGR TMEV software with a median number of false significant genes set at 0. Note the large shift of the distribution to the right with increasing phylogenetic distance. C) Hierarchical tree of all CGH arrays, in a multiclass analysis using the Euclidean distance metric with average linkage clustering [82]. Arrays from the same species are clustered together, and the hierarchy is consistent with phylogenetic species relationships. The autologous hybridization (zebra finch – zebra finch, not shown) is effectively the outgroup in this analysis. D) The distribution of log2 ratios (test species:zebra finch reference) for one spot across 28 arrays (7 species).

A clear effect of phylogenetic distance is evident in the cross hybridizations to the zebra finch array using DNA from the kingbird (suboscine) and the chicken (non-oscine), as shown by histogram analysis log(2) ratios for all the spots on the arrays (Fig. 3A). Histograms from hybridizations using genomic DNAs from the other oscine species (not shown) were essentially indistinguishable from the zebra finch histogram when all spots on the array were included. However, statistical analysis detected a small subset of spots with significantly different hybridization signals in the other oscines relative to the zebra finch. Histograms focusing on the 200 genes showing the greatest effect of species are presented in Fig. 3B. With the three other Passeroidea (canary, song sparrow, white crowned sparrow), the histogram peak height is reduced somewhat (note change in Y-axis scale) and a scattering of genes appears with log(2) ratios of 1–3 (zebra finch to test species). With the somewhat more distant starling the number of genes with reduced signals is slightly larger, and the number larger still with the kingbird DNA. With the chicken DNA, the central peak (log2 = 0) is abolished altogether and the curve for these 200 genes is now shifted by approximately 2 logs to the right. Hierarchical clustering of the CGH array data is also consistent with the general phylogenetic relationships of the species under comparison (Fig. 3C).

Interestingly, we also noted cases where particular spots on the zebra finch array gave strongest signals against the genomic DNA of species other than the zebra finch (e.g., negative log values in Fig. 3B). An example is shown in detail in Fig 3D. Note the sharp drop in signal intensity against all species other than zebra finch except for the starling, where a sharp increase in hybridization signal is detected. We speculate that cases like this may represent species differences in gene copy number.

### The Community Collaborations

The production of the SoNG 20 K array resulted in enough PCR product to print at least 1000 microarrays. To engage the broad songbird research community in this research while maintaining consistent technical standards, we devised the following plan. In 2004 we circulated a broad invitation for investigators to apply for use of some of the resulting DNA microarrays. We offered to perform the RNA extractions and hybridizations centrally by a single skilled investigator (KR), at no cost to the applicant beyond the cost of producing the tissue. Each initiating investigator would have control over initial publication of resulting data, but all data would eventually be available for use in a planned meta-analysis. The Call for Applications is provided here as Additional File 2. In response, 16 research groups proposed a set of 25 experiments representing a range of research questions and foci (Table 7). In March 2005, these proposals were reviewed by the central steering committee for the SoNG Initiative [8], with feedback offered as appropriate to the initiating investigators. Systematic execution of these experiments began soon thereafter and should be completed by the end of 2008.

**Table 7:** Proposed "Community Collaborations" for songbird microarray studies.

				**Primary Contrast (question)**				
				
**#**	**Locale**	**Group**	**Species**	**1**	**2**	**3**	**4**	**5**
1	AZ	A*	Zebra finch			•		
2	WA	B	White-crowned sparrow					•
3	NY	C*	Zebra finch			•	•	
4	NY	C*	Zebra finch			•	•	
5	Hungary	D*	Long-tailed, great & penduline tit	•	•			
6	CA north	E*	House finch, red crossbill	•				•
7	MI/CA	F	Zebra finch		•	•		
8	MI/CA	F	Zebra finch		•			
9	MI/CA	F	Zebra finch		•			
10	MI/CA	F	Zebra finch		•	•		
11	Netherlands	G*	Zebra finch				•	
12	AZ (Phoenix)	H*	House finch					•
13	AZ (Tempe)	H*	White-crowned sparrow					•
14	IN	I*	White-throated sparrow	•	•			
15	FL	J*	Zebra finch			•	•	•
16	MD	K	European starling					•
17	OR	L	Zebra finch	•			•	
18	OR	L	Parrot, hummingbird				•	
19	OR	L	Zebra finch				•	
20	US West	M*	Thrush, Junco, House sparrow, Cordillerian flycatcher	•				
21	IL	N	Zebra finch				•	
22	IL	N	Zebra finch			•	•	
23	IL	N	Zebra finch				•	
24	IL	O	Zebra finch			•		
25	WA	P*	Song sparrow	•				•
CGH	14 non-Tgu species proposed							

Experiments (identified by # in first column) were nominated by investigators in the community following specific guidelines (Additional File 2). Investigator groups are indicated by Locale and a "group" letter code (columns 2 and 3); asterisk indicates investigators outside of the core group responsible for program development. Experiments were classified according to the Primary Contrast (question) defined by the experimental design, with these general contrasts as indicated by number in the table: 1, Comparative; 2, Brain Sex; 3, Critical Period; 4, Activational; 5, Environmental. CGH: Comparative Genomic Hybridizations validating zebra finch array use against the 14 other species proposed for study across all experiments.

## Discussion

Using a pipeline for high-throughput sequencing and bioinformatics, we generated ~72,000 reads of sequences expressed in the zebra finch brain. We incorporated these and 27,000 more reads from other projects in the community into a single master database available to the public via the internet [9]. Our approach has been iterative, progressing through three sequential builds each one incorporating additional data. We used our evolving sequence assemblies in the design of two different microarrays for gene expression studies and we have deployed these resources in a large set of collaborations that should continue to bear fruit in the future.

The 86,784 filtered high-quality sequences in the current (third) build of ESTIMA:Songbird describe approximately 31,658 non-redundant transcript segments. Although this is a dramatic increase in the number of sequence information available (there were only 72 records for songbirds in Genbank when our project began [36]), this collection is still only a partial representation of the sequence diversity in zebra finch brain. Most of the sequences are represented by only a single read and even after three library subtractions, one of every three new sequences generated was still novel. An early analysis of RNA complexity in the canary forebrain suggested a population of as many as 100,000 different transcripts, with total nucleotide diversity approaching the size of the genome itself [37]. This complexity seemed implausible when that analysis was first done, but recent studies in species from flies [38] to humans [39-41] have now demonstrated that transcription is much more widespread in the genome than previously anticipated.

Some of the 31,658 non-redundant sequences undoubtedly represent discontinuous portions of common transcripts, including alternative splicing products as well as non-overlapping ESTs. Indeed, using the chicken IPI annotation, we detected significant matches for 13,219 sequences but only 8127 (61%) of these IPI identifiers were unique. Presumably this indicates that a substantial fraction of the 31,658 "unique" sequences in the SB3 assembly are in fact derived from a smaller set of genes; by extrapolation this suggests an upper bound of 19,311 (31658 × 61%) unique transcription units represented by the SB3 assembly. Hence we have no reason at this juncture to expect the complement of protein coding genes is any larger for the zebra finch than it is for the chicken, i.e., 20,000–23,000 [33].

Approximately 23% of ESTIMA:Songbird 3 sequences do not align significantly against the current build of the chicken genome (WASHUC2, May 2006). Some of these could represent orthologs of sequences that are present in the chicken genome but not yet incorporated into the chicken genome build. The current chicken genome assembly is believed to lack approximately 8% of the genes in the full genome complement, including sequences on the female-specific W chromosome in particular. Our cDNA libraries were prepared from a mix of both sexes.

As is true for all gene indices, annotation of function is a major ongoing challenge. Very few genes in songbirds have received any sort of direct experimental analysis thus we must rely on inferences based on similarities to sequences in other species. The closest species with significant genome annotation is the chicken. However, annotation of the chicken genome itself has relied heavily on putative orthologies to mammalian sequences. During our three sequential builds we probed a number of databases to assess their utility for generating putative functional annotations according to sequence similarities. As of Build 3, we focused on the chicken International Protein Index as a primary annotation reference. This is a relatively conservative reference and reflects alignments only with known or predicted protein coding sequences. Only 42% of our Build 3 assembled sequences align against the IPI index, and these represent only a third of the total number of sequence identifiers in the complete chicken IPI index. We anticipate that many of the currently unannotated Build 3 sequences probably represent non-coding segments of IPI-annotated genes. Identification of these relationships should follow from the annotation of the zebra finch genome assembly, expected in 2008.

Our database probably also contains some non-coding RNAs [42]. A preliminary BLAST analysis of the 86,784 ESTs in our current database against miRBASE [43], a compendium of ~5000 known microRNA sequences, revealed significant alignments for 789 zebra finch ESTs against 489 mature microRNA sequences (data not shown). Additional work will be required to establish whether these sequences are indeed processed into mature microRNAs in the zebra finch.

To stimulate broad application of genomic approaches to songbird research, we generated a cDNA microarray from our first EST build and organized a Community Collaboration system for design and execution of experiments using this array. Part of our group also developed an Affymetrix oligonucleotide array based on our second EST build [15]. To validate these tools and to refine the general methods for the Community Collaborations we did several analyses. These analyses included assessment of optimal methods for RNA purification, MA plots of hybridization data, amplification and labeling from dissected brain samples; and methods for microarray statistical analysis. Some of these studies are summarized in our Call for Community Collaborations (Additional File 2). Validation of the Affymetrix array has been done by the group at Lund University and is not included in this publication. In our main report here, we described several analyses that may have more general implications for transcriptome analysis in songbirds.

The SoNG 20 K cDNA microarray includes 5 different probes for transcripts derived from the *zenk *gene, including one from the canary sequence. All probes gave signals well above background with labeled cDNA from the dissected auditory lobules of individual birds hearing either song or silence, and all reported an increase in signal in the song-stimulated birds relative to the silence controls. Comparing the near-full positive control probes from zebra finch and canary, both gave equivalent signal intensities and fold-change measurements. In the direct-comparison design used for this experiment, a group size of 6 birds per condition hybridized on 6 arrays was sufficient to reach fairly robust statistical significance for three of the single-spotted probes. The fourth (SB02047B2F12.f1, the 5'-most EST) would not have passed the threshold for identification using even generous criteria (e.g., raw p < 0.05, FDR < 20%). This EST reported a mean intensity that was almost three-fold higher than for the zebra finch and canary positive control probes. We suspect this may indicate cross-hybridization to some other sequences for this particular probe. Alternatively, the variations in mean intensities and fold-changes for the five different probes could indicate differential transcription or RNA processing across the transcript, phenomena that are increasingly observed in high-resolution analyses of transcription units [40]. It is also worth noting that in this preliminary experiment we detected changes in 220 other probes at the significance threshold of the single-spotted zebra finch cDNA postive control (FDR 16%). Analysis of song-regulated sequences is a topic of several of the proposed Community Collaborations and will be described in detail elsewhere.

With the production of the Lund-zfa array after ESTIMA Build 2 [15], we were able to compare hybridization results using the same tissue samples on the two array platforms. Almost all of the transcripts represented on each platform were detected in adult male and female brains, and there was a general concordance between the platforms on the estimated log2-fold changes for the transcripts they had in common. While the exact "significant" gene lists varied slightly, much of this difference is probably due to differences in sample treatment and analyses between the two array types, which determine the probability of detecting differences. For, example, the SoNG-20 K arrays used a common reference design which helps to normalize differences between arrays, whereas the individual samples had additional shipping and handling before being hybridized to the Lund-zfa arrays, which could have added to their variability. Despite the differences between the platforms, a principle components analysis (Fig. 2B) shows that the biological differences between males and females dominate over any technical differences between the arrays. In sum, both array platforms describe a similar overall picture of differential gene expression and should be useful for further studies.

When we surveyed the songbird research community for interest in using the 20 K array, we recognized that there was considerable interest in experiments involving species other than the zebra finch (Table 7). We initiated comparative genomic hybridizations at this point primarily to evaluate the feasibility of cross-hybridizations with other species. The use of single-species microarrays for species cross-hybridizations is a controversial point in the literature (e.g., pro: [44-50]; con: [51]; cautionary: [49,52,53]). Direct comparison of gene expression in different species on a single-species array is especially problematic, as variations in gene copy number, sequence divergence and RNA expression levels are all confounded. However, for most of the experiments initially proposed for Community Collaborations, the goals were to analyze particular phenomena within a single species, focusing on phenomena that are not well represented in the zebra finch (e.g., effects of photoperiod). We believe that our cross hybridization studies clearly validate our 20 K cDNA microarray for within-species comparisons of other oscine songbirds (Fig. 3), with the caveat that some minority of the probes will give reduced signals compared to hybridizations with zebra finch material. One must also interpret any annotations with particular care, and cross-hybridization results based on particular zebra finch array probes may need to be verified independently, e.g., by resequencing in the target species and RT-PCR to confirm regulation.

The Lund-zfa array was developed with our second build of ESTIMA:Songbird (zebra finch), and it was specifically designed with the intent of supporting research in other songbird species. Quantitative analysis of CGH to DNA from the common whitethroat (*Sylvia communis*; family Sylviidae) supports the efficacy of cross-hybridization as 96% of the ESTs are called as present [15]. Taking these studies together with our own, zebra finch probes have now been shown to be adequate for detecting signals in the all three major superfamilies of the Passerida parvorder of the oscines (divergence time < 50 MYA) [1,54,55]. However, our data for the kingbird (Fig. 3) suggest that use of zebra finch arrays for analysis of sub-oscines (divergence time ~70 MYA) may be more problematic. Direct empirical tests are needed to establish viability for use with Corvid species, oscines that diverged from the Passerida ~50 MYA.

## Conclusion

With an inclusive and integrative approach, the Songbird Neurogenomics Initiative has established a rigorous foundation for application of genomic technology by the community of researchers focused on songbirds. Already our ESTIMA:Songbird database has been used in the study of gene dosage compensation in birds vs. mammals [56], sex chromosome evolution [57], regulation of brain caspase-3 activity in experience-dependent plasticity [58], development of markers for study of population genetics in zebra finches [59], and identification of sequences subject to positive selection in birds (Axelsson et al., submitted). With the third build of the ESTIMA:Songbird database now complete, we anticipate future improvements in transcriptome annotation will soon come from other new approaches including whole genome tiling arrays and massively parallel sequencing [60,61]. In the meantime, the collection of microarray experiments organized here (Table 7) will generate a broad profile of gene expression in the songbird brain across multiple dimensions of species, sex, neuroanatomy, developmental age, physiological state and behavioral context. The active engagement of a broad research community was instrumental in the selection of the zebra finch as the second avian species for whole genome sequencing, after the chicken [62]. Continuing community participation will be crucial in applying these emerging resources to the expanse of fundamental research questions posed by songbirds.

## Methods

### Sources of cDNA Sequences

Clones for sequencing were drawn first from two normalized libraries (designated SB01 and SB02) representing zebra finch brain RNA from both sexes at multiple ages. Three generations of subtracted libraries (SB03, SB05, SB06) were then prepared sequentially from SB02, by removing the clones sequenced in the previous rounds using PCR subtractive hybridization. For clones from these libraries, sequence reads were generated from the 5' end of each insert. Additional sequence records were also obtained from colleagues at Duke University [63] and Rockefeller University. Details for each cDNA source follow.

#### SB01

2,880 cDNAs were donated by participant Juli Wade, from a normalized library produced at Michigan State University representing d10-60 male and female telencephalons [64]. Average read length was ~510 nucleotides, and sequence redundancy was 9%.

#### SB02

A new normalized, directional library [65] was produced for this project using zebra finch brains contributed from aviaries at UCLA (A. Arnold), Oregon Health and Science University (C. V. Mello), Michigan State University (J. Wade) and University of Illinois (D. Clayton). The collected tissues represented telencephalon of both sexes at each of 5 ages: adult, day45, day10, day1 and embryonic. Total RNA was extracted separately from each of the 10 groups, using the Trizol method, and pooled in approximately equal amount. The integrity of total RNA was verified by denaturing agarose gels and by spectrophotometry (ratio A_260/280_). Poly(A)^+^mRNA was isolated twice from total RNA using the Oligotex Direct mRNA kit (Qiagen). mRNA was reverse transcribed into double stranded cDNA using a modified oligo_18_(dT) primer with an identifying tag sequence. Double stranded cDNAs were size selected (more than 450 bp). Size selected cDNAs were adaptored with EcoRI adapters at both ends and digested with NotI. The cDNA was then directionally cloned into EcoR1-NotI digested pBluescript II SK(+) vector (Stratagene). Purified plasmid DNA from the primary library was converted to single-stranded circles and used as a template for PCR amplification using the T7 and T3 priming sites flanking the cloned cDNA inserts. The purified PCR products, representing the entire cloned cDNA population, were used as a driver for normalization. Hybridization between the single-stranded library and the PCR products was carried out for 44 hours at 30°C. Unhybridized single-stranded DNA circles were separated from hybridized DNA rendered partially double-stranded and electroporated into DH10B cells to generate the normalized library. The normalized library had a total of 4.6 × 10^6 ^colony forming units (cfu) with less than 1% empty vectors (blue colonies). Average insert size, as determined by PCR of random clones, was 1 kb. Average sequencing read length was ~710 nucleotides. After 18,720 reads, redundancy was 41%.

#### SB03

A subtracted library was produced from SB02 by removing previously sequenced clones as described in [65]. The total number of clones of the SB03 library was 3 × 10^6 ^cfu. SB03(a): For ESTIMA:Songbird Build 1, 19,584 clones were sequenced from the subtracted library, with an average clean read length of 700 nucleotides. SB03(b): For ESTIMA:Songbird Build 2, an additional 9,120 sequences were determined.

#### SB05

a second round of subtraction was performed in which clones sequenced from the SB02 and SB03 libraries were removed from the SB03 library as described [65]. The resulting library was named SB05 [there is no SB04 – that designation was used in quality control procedures]. The titer of the subtracted SB05 library was 4.6 × 10^6^. A total of 11,328 clones were sequenced from this library with an average clean read length of 812 nucleotides.

#### SB06

the third round of subtraction involved the removal of all sequenced SB02, SB03 and SB05 clones from the SB05 library. The new SB06 subtracted library had a titer of 2.2 × 10^6^. An additional 11,040 clones were sequenced from SB06, with an average clean read length of 635 nucleotides.

#### Duke sequences

Sequence data for was obtained directly from Erich Jarvis and colleagues at Duke University [14]. Reads had been performed from both 5' and 3' ends of each cDNA. For Build 2, we obtained the raw sequencing chromatograms and processed them as for the SB library reads at Illinois. 64% (23,225) met the minimum quality criteria. For Build 3 we used the final sequence records (17,312) as deposited in NCBI by the Duke investigators, where each record represents a single unconnected read from one end (either 5' or 3') of a single clone. In ESTIMA:Songbird, these sequences are identified by their Genbank IDs, which begin with initial letters "DV".

#### Rockefeller sequences

Sequence data as deposited in NCBI was obtained directly from XiaoChing Li at Rockefeller University [16]. In ESTIMA:Songbird, these sequences are identified by their Genbank IDs, which begin with initial letters "EE".

### High-Throughput Sequencing

For high-throughput sequencing from the cDNA libraries described above, bacteria were plated on agar plates with 100 μg/μl of carbenicillin and colonies were robotically picked with the Genetix Q-pix and racked as glycerol stocks in 384-well plates. After overnight growth of the glycerol stocks, bacteria were inoculated into 96-well deep cultures with 0.7 ml of Luria Broth and 100 μg/μl of carbenicillin and grown for 18 hours. Plasmid DNAs were purified from the bacterial cultures with the Qiagen 8000 and Qiagen 9600 BioRobots. Sequencing reactions were set up as follows: 4 μl of 12.5% glycerol, 2 μl of 5X sequencing buffer (Applied Biosystems), 1.5 μl of 1 μM T7 standard primer, 1 μl of H_2_O, 0.5 μl of BigDye terminator (Applied Biosystems) and 1 μl of template. Thermal cycling was performed at 96°C for 5 min followed by 35 cycles of 96°C for 15 sec, 53°C for 5 sec and 60°C for 4 min. Reaction products were precipitated with 70 μl of 0.2 mM MgSO_4 _in 70% ethanol. Samples were resuspended in 10 μl of formamide (Applied Biosystems). Sequencing reactions of the 5'ends were performed on ABI 3730 × l capillary systems. All 384- and 96-well format plates were labeled with a barcode and a laboratory information management system (HTLims) was used to track sample flow.

Base-calling was performed using the Phred program. Vector sequences were trimmed using Cross-match. Low-quality bases (Phred quality score < 20) were trimmed from both ends of sequences using Qualtrim and Simpletrim software developed at the W. M. Keck Center (University of Illinois). Those ESTs having a length of more than 200 base pairs after both vector and quality trimming were considered "high-quality" ESTs. The repeat sequences in these ESTs then were masked by the RepeatMasker program using vertebrate repeat sequences database as reference. The masked sequences were then searched for sequences from bacterial chromosomal DNA, RNA, viral DNA, rRNA, and mitochondrial DNA using BLASTN).

### EST Assembly

Paracel Transcript Assembler (based on CAP4) was used to assemble the ESTs in a two-stage process [66]. First, related sequences were clustered using BLASTN. Each cluster was then assembled into contigs where possible (i.e., no discontinuities in the alignment). All BLAST searches were conducted using the NCBI stand-alone BLAST software. The maximum E-value for an alignment to be judged significant was 10^-5^, an arbitrary value precedented by other studies [11-13]. The nonredundant EST set was searched by BLASTx against the NCBI nonredundant protein database (NR.aa) and SWISSPROT. Statistics for the three sequential assemblies are given in Table 1. For example, for ESTIMA:Songbird Build 1, 87% of SB01, SB02 and SB03(a) reads passed all criteria above and were included as "filtered, high quality sequences," for a total of 35,232 sequences in the assembly.

### EST Annotation

BLASTn was used to search against the chicken genome sequence (Gga genome) assembly (WASHUC2, May 2006) [67] and tBLASTx to align against databases representing chicken transcripts in NCBI (Gga Unigene build 33, NCBI Chicken RNA downloaded May 17, 2007), Ensembl (Chicken cDNA-All and Chicken cDNA ab initio, downloaded May 17, 2007), and the archive [68] of the TIGR Chicken Gene Index GGGI release 11 [69]. We also used tBLASTx to search against the Human (Hs) Unigene database (Build 201), the NCBI Chicken protein database (downloaded May 17, 2007) and the Chicken International Protein Index (IPI, Release 3.26). Default parameters were used for all BLASTs, which were performed locally with TimeLogic Server. The maximum E-value for an alignment to be judged significant was 10^-5^, an arbitrary value precedented by other studies [11-13]. Where multiple targets within a database produced alignments with significant E-values, the single best-scoring alignment was saved. Descriptive details for each alignment are available for each record in the ESTIMA:Songbird database [9]. To obtain a short, descriptive working name for annotated sequences, where available we imported the IPI protein name into our ESTIMA database in the "custom annotation" field.

### Production of 20 K spotted cDNA Array

Individual cDNA clones were selected to represent each of the 17,878 unique sequences in the primary assembly. For contigs that comprised multiple clones, we selected a single clone for the microarray as follows:

a. for each EST, we compared top 5 hits against NCBI(NR), vs top 5 for whole contig consensus sequence; eliminated ESTs having no correspondence to any of the contig's hits [rationale: their inclusion in the contig could be an artifact – direct similarity to the core conserved consensus is validating].

b. selected the EST farthest upstream ["5'-most," should be the longest, and the most useful for cross-hybridizations]

c. where multiple ESTs shared the 5'-most ends, we picked the longest.

d. for the minority of contigs that did not align against NR, we found the longest ORF in the contig consensus and selected the EST that gave the best match (by TBLASTN) to the consensus ORF.

The cDNAs representing these clones and controls were rearrayed in 384 well plates with a Genetix QPix clonepicking instrument and stored as a stock clone set at -80°C. Sample clones from each plate were resequenced for quality control and accuracy of clone transfer. Stock clones were inoculated for overnight cultures in LB media containing 75 μg/ml ampicillin and 8% glycerol. 7.5 μl diluted (1:14) culture was added to 75 μl PCR reactions containing 37.5 μl custom 2X Platinum PCR supermix (Invitrogen, Carlsbad, CA), 0.3 μl of 100 μM forward and reverse primers (representing the vector sequence flanking the cDNA inserts). PCR parameters were as follows: Initial denaturation at 95°C, 10 min. followed by 35 cycles of 95°C, 40 sec., 65°C, 40 sec, 72°C, 3 min, and a final extension at 72°C for 5 min. PCR products were checked by electrophoresis on E-gel 96 1% gels (Invitrogen, Carlsbad, CA). Failed reactions were reamplified as above with the addition of 1 M Betaine to the PCR reaction. PCR product was cleaned with Millipore PCR 96 cleanup plates (Millipore, Billerica, MA). All liquid manipulations were carried out in 96 well format on a Beckman Biomek FX (Beckman Coulter, Fullerton, CA). Cleaned product was transferred to 384 well plates, dried in a speedvac and resuspended in 3 × SSC and 1.5 M Betaine buffer for printing. using a GeneMachines OmniGrid 100 robotic printer onto Corning GAPSII amino-silane coated slides. PCR product sufficient for printing 1200 arrays was generated during July-September, 2004.

### Song stimulation

All procedures involving animals were conducted with formal institutional (IACUC) approval and oversight. For the microarray analysis, male zebra finches 3–17 months old (age-balanced, two groups) were hatched and reared (3 birds/cage; 12:12 light:dark cycle) in D. Clayton's aviary at the Beckman Institute, Urbana IL. During 2/20/2005–3/10/2005, each was individually isolated in a sound attenuation chamber for 46 hours, then exposed to a 30 minute song playback (or silence) between noon and 1 PM. Behavioral responses were videotaped for subsequent quantification of a "listening" index [70]. At the end of the 30 minute stimulation period, the birds were immediately euthanized by decapitation, and the two auditory lobules (AL) were dissected out [71], put into RNAse-free 1.5 ml tubes and frozen on a dry ice/ethanol mixture. Tissue was stored at -80°C until RNA preparation.

### Microarray hybridization (Song stimulation experiment)

Total RNA was prepared with RNAqueous-Micro kit (Ambion; average yield = 4 μg/AL-pair). 500 ng RNA was amplified using the Low RNA Input Fluorescent Linear Amplification kit (Agilent; average yield = 25 μg). The resulting aRNA was reverse transcribed using an indirect aminoallyl incorporation protocol and labeled with either Cy3or Cy5 dyes (GE Healthcare). Dye labeling was balanced by group (i.e., half of each group, song or silence, was labeled with Cy3 and the other half with Cy5). Six arrays were hybridized simultaneously, each array with one Song and one Silence sample. Thus each array represents one of six biological replicate measurements of the song-vs-silence relationship for each spot on the array. Slides were hybridized overnight at 42°C, washed and scanned using an Axon GenePix 4000B microarray scanner. All slide images were analyzed using GenePix Pro 6.0 software. Analyzed slide images were manually edited and aberrant spots were flagged for exclusion in downstream analysis.

### Statistical analysis of hybridization data (Song stimulation experiment)

Prior to analysis, the fluorescence intensities were edited by removing automatically and manually flagged spots that did not surpass minimum quality thresholds. "Background" was defined independently for each spot from its surround and subtracted from each spot value. The data were log2 transformed and loess normalized within each array. Analysis then proceeded using the two-step approach of Cui et al. [72], where the normalized data were first adjusted for global array and dye effects across genes, then the resulting adjusted intensities were analyzed by gene with a parametric linear model [73-76]. This model was implemented using Bioconductor R-routines [77] and the MIXED procedure (SAS, 2004).

For the analysis of the five different *zenk *sequences (Figure 1, Table 6), replicate spottings of the same cDNA on one array (e.g., the redundant spottings of the canary *zenk *cDNA) were treated as replicate measurements, whereas cDNAs representing different segments of the same gene were treated as independent targets (e.g, each of the other four zebra finch *zenk *cDNAs spotted once per array).

### Production of Universal Reference RNA sample

Total RNA was prepared from whole telencephalon of 30 birds [3 males and 3 females from each of the aviaries of Clayton, Wade, Arnold, Mello, and D. Perkel (University of Washington)] using TRI Reagent (Ambion). The total RNA was DNase I treated (Turbo DNase, Ambion) and cleaned up on a spin column (RNeasy, QIAgen). Equal amounts of RNA from each bird were pooled. Aliquots of the pool were amplified (Low Input Linear RNA Amplification Kit, Agilent) and pooled. Sufficient cRNA for 5× more arrays than necessary was generated and stored at -80 in small aliquots. A test hybridization of the reference sample, labeled only with Cy3 (GE Healthcare), showed signal detectable above background on greater than 95% of probes.

### Concordance of SoNG 20 K and Lund-zfa arrays

RNA samples were prepared from 11 individual zebra finch brains (5 adult males, 6 adult females) and hybridized to SoNG 20 K arrays as described above for the Song Stimulation experiment, except that a common reference design was used. One male or female sample was hybridized to one channel of an array and the universal reference sample was hybridized to the other channel of each array. No technical dye-swaps were done; instead possible dye-bias was controlled by putting the reference sample in the Cy3 channel for ~ half the male and ~ half the female samples and in the Cy5 channel for the other half of the samples. After the analysis in the US the samples were kept at -80°C for 2 months before they were shipped on dry ice to an Affymetrix service provider in Sweden, the Swegene Center for Integrative Biology at Lund University (SCIBLU MARC) [78], where hybridizations to the Lund-zfa array were performed. The experiments in the present study used the initial version of the Lund-zfa array which was an Affymetrix design spotted with NimbleGen technology [15]. (Note that since then, the Lund-zfa Array has been re-designed and the version currently distributed is spotted like any Affymetrix standard array.). Before hybridization the RNA samples were quality checked using a Nanodrop spectrophotometer and RIN values were calculated using an Agilent 2100 Bioanalyzer. One high quality sample representing each of the 11 individuals was hybridized according to standard Affymetrix protocols for RNA. Five μg total RNA from each sample was used in the regular protocols for GeneChip^® ^Arrays and hybridized onto the Lund-zfa Affymetrix array overnight in the GeneChip^® ^Hybridisation oven 6400 using standard procedures. The arrays were washed and then stained in a GeneChip^® ^Fluidics Station 450. Scanning was carried out with the GeneChip^® ^Scanner 3000 and image analysis was performed using GeneChip^® ^Operating Software.

Pre-processing of each array type was done in R using Bioconductor packages appropriate for spotted or Affymetrix arrays. Because > 98% of spots on the SoNG 20 K arrays were above local background estimates, no background correction was performed. Within-array printtip loess normalization was performed, as was a between-array scaling normalization using the limma package [79]. For the Lund-zfa arrays, pre-processing was done using RMA (with the normal background correction included) [80]. The Lund-zfa array is a PM only array, with Perfect Match probes arranged in a checkerboard pattern on the array and 4 empty features adjacent to each PM probe [15]. We used the signals from the 2% lowest empty features on the chip (5111 features distributed throughout the spotted area) + 2 standard deviations as an estimate of the global background of each chip, and this background cut-off was then used to calculated the percentage of present call probes. To use the 2% lowest signals is a standard Affymetrix cut-off for background calculation. Both array types were analyzed for differential expression using the limma package, which fits a mixed linear model with an empirical Bayes error correction. Multiple test correction of the p values was done using the false discovery rate (FDR) method [81].

### Comparative Genomic Hybridization (CGH)

Tissue or blood was obtained from two males and two females of each species. Genomic DNA (gDNA) was collected from each sample using the GenomicPrep Cell and Tissue or Blood DNA Isolation Kit (GE Healthcare). gDNA was digested with Hae III for 5 hours at 37°C and cleaned using a Qiaquick PCR purification kit (QIAgen). A reference sample was created by pooling equal amounts of digested and cleaned DNA from 2 male and 2 female zebra finches. Template DNA was polymerized from 4 μg digested and cleaned DNA using high concentration Klenow (New England Biolabs) and incorporating amino-allyl dUTP (Sigma Aldrich) for 4 hours at 37°C. The resulting amino-allyl labeled DNA was coupled with Cy dye (GE Healthcare), cleaned, and hybridized using SlideHyb #1 hybridization buffer (Ambion). All experimental samples were labeled with Cy3 and all reference samples were labeled with Cy5. Slides were hybridized for 48 hrs at 42°C, washed and scanned using an Axon GenePix 4000B microarray scanner. All slide images were analyzed using GenePix Pro 6.0 software. Analyzed slide images were manually edited and aberrant spots were flagged for exclusion in downstream analysis. Normalization and statistical analysis [82] was performed using the TM4 software suite developed by TIGR. Slides were within-slide LOWESS normalized using MIDAs.

### Availability

All derived sequence resources are publicly accessible via internet at the Songbird Neurogenomics website [8], which includes a link to the ESTIMA:Songbird EST database [9]. Resources include raw sequencing chromatograms, contig alignments, text files of both raw and trimmed sequence, BLAST searching of the database, a GO Browser, and annotations imported from BLAST searches against the databases listed in Table 3. All ESTs have been deposited in Genbank (Accession numbers DV944971 - DV962014, CK301200 - CK317559 and FE712085 - FE739917). It should be noted that the Genbank records are restricted to "high quality" trimmed sequence data, which typically represents only a portion of each cDNA spotted on the array. For access to the Duke-derived annotations of their cDNAs, we incorporated direct links to the Duke website [63] in the records presented on the ESTIMA:Songbird website. Clones in the ESTIMA database can be purchased through the Clemson University Genomics Institute (CUGI) [83] distribution service. Raw microarray data is archived and distributed in MIAME-compliant form using 3rd Millenium's ARDAS (Array Repository and Data Analysis System) server [84] and is accessible using "public" (no quotes) as the login and password.

## Abbreviations

CGH: Comparative Genomic Hybridization; AL: auditory lobule of the caudomedial telencephalon; NCM: caudomedial nidopallium; GWCS: Gambel's white-crowned sparrow (*Zonotrichia leucophry gambelii)*; SoSp: Song sparrow (*Melospiza melodia)*; KingB: Western Kingbird *(Tyrannus verticalis)*; Can: Canary *(Serinus canaria*); Star: European starling (*Sturnus vulgaris)*; Chick: Chicken *(Gallus gallus)*; G.Ga: *Gallus gallus *(chicken); T.gu: *Taeniopygia guttata *(zebra finch); EST: Expressed Seqence Tag; SAM: Statistical Analysis of Microarrays; MYA: Million Years Ago; gDNA: genomic DNA; FDR: False Discovery Rate; UIUC: University of Illinois at Urbana-Champaign; SoNG: Songbird NeuroGenomics

## Authors' contributions

KR: PCR amplification of DNAs for microarray production, tissue RNA extraction, genomic DNA extraction, probe labeling, microarray hybridizations, initial Genespring analyses, Community Collaboration liaison.

AA, GB, EB, JG, CM, JW and DFC: founding members of the Songbird Neurogenomics Initiative, responsible for overall project planning, review of results, and generating grant support.

MB: microarray production in the Keck Center

SB, DH, SN: producers of the Lund-zfa array

SD: designed and executed the song stimulation experiment and independently verified the statistical analysis of results.

JD: statistical comparison of Lund-afa vs. SoNG 20 K cDNA hybridization data

MF: analysis of CGH data (Fig. 3).

AH: cDNA library production in the Keck Center

RK: high-throughput sequencing in the Keck Center

GG, LL, JT: CAP/PTA Assembly, BLAST analyses and ESTIMA database development and maintenance in the Keck Center.

HL: founder of the Keck Center and general advisor to the project.

PL: designed and performed the BLAST analysis in Table 2.

SRZ: designed and executed the mixed-model analysis used in Table 6 and as the template for Community Collaboration analyses.

DFC: principal investigator of the Songbird Neurogenomics Initiative grant project; coordinated the project as a whole and wrote the manuscript with contributions from all of the other authors.

All authors read and approved the final manuscript.

## Supplementary Material

Additional File 1 SoNG20K. Annotation of the SoNG 20 K array. standard GAL file format describing each spot on the 20 K microarray, 20215 rows × 6 columns. Rows 1–55 describe block organization. Rows 56-20215 describe the 20160 features (spots) on the array (see Table 4). Columns 1–3 define feature position. Column four gives the unique identifier for the feature. Column five gives the "custom annotation" name for that spotted DNA, based on the Build 3 annotation (described in Tables 3). Column six identifies the source of the information used for that custom annotation. Click here for file

Additional File 2 SoNGcall. CALL FOR PROPOSALS for USE OF MICROARRAY RESOURCES. Document distributed on Oct. 15, 2004, describing the overall goals and administrative organization of the Songbird Neurogenomics Initiative. Click here for file
