# Supplementary material for: The Songbird Neurogenomics (SoNG) Initiative: Community-based tools and strategies for study of brain gene function and evolution
Source: BMC Genomics. 2008 Mar 18;9:131. doi: 10.1186/1471-2164-9-131 (PMC2329646; doi:10.1186/1471-2164-9-131)
Supplement: Additional File 2 — SoNGcall. CALL FOR PROPOSALS for USE OF MICROARRAY RESOURCES. Document distributed on Oct. 15, 2004, describing the overall goals and administrative organization of the Songbird Neurogenomics Initiative. [file 1471-2164-9-131-S2.pdf]

CALL FOR PROPOSALS for USE OF MICROARRAY RESOURCES

Songbird Neurogenomics (SoNG) Initiative

URL: http://titan.biotec.uiuc.edu/songbird/
David F. Clayton, PI. (dclayton@uiuc.edu)
October 15, 2004

I. Introduction

New resources are now available to qualified researchers interested in measuring brain gene expression in songbirds. These resources are made available with the support of NIH grant NS045264 ("Songbird Neurogenomics Initiative"), which represents a working group of more than a dozen scientists at six institutions (PI: David Clayton, University of Illinois). This group, referred to here as the **SoNG Initiative**, is producing the following community resources:

- A keyword-searchable database of more than 34,000 Expressed Sequence Tags (ESTs) derived from zebra finch brain. This database is already open to the public and may be used accessed freely via the internet.
- A DNA microarray containing probes for ~18,000 different genes expressed in the songbird brain;
- A collaborative infrastructure for planning and executing DNA microarray experiments.
- An internet-accessible archive of data resulting from experiments using these microarrays.

To make specific use of the new microarray resources, investigators have two options, as developed in detail in the rest of this Call for Proposals:

1) propose a specific experiment to be carried out as a collaboration with investigators in the SoNG Initiative. The Proposer will be expected to provide biological material (e.g., dissected brain samples), but costs of microarrays, hybridizations and data analysis will be borne internally by SoNG Initiative core support mechanisms.
2) apply for direct purchase of microarrays, without formal collaborative involvement of the SoNG Initiative. Investigators will not be subjected to any formal review of experimental plans, nor will have any restrictions on their use of the arrays, save for a single provision: they must provide resulting microarray hybridization data, in timely fashion, to the publicly accessible data archive. (This is now a condition for publication of data in all of the leading journals).

For either option, investigators must submit an application to the SoNG Initiative by **January 15, 2005,** to receive full consideration. This document provides further detail about the background and organization of the SoNG Initiative, the specific resources available, the process for applying for use of these resources, and the review process for considering such applications.

II. Background

Potential Impact of Genomics on Songbird Research

High-throughput genomic technology is transforming the way biological science is done. Instead of focusing on a single gene or protein (often with the idea that it may be a "model" for some general process), one can now obtain comprehensive readouts of literally thousands of gene products in a single experiment. This is especially important for the science of brain and behavior, where important phenomena typically have multigenic influences and are not simply determined by a single gene product.

Genomic technology is changing science in another way, by driving the development of large, community-based research teams. At a practical level, no single laboratory typically has the resources to sequence a genome or develop a large microarray. However, these resources are now easily within the grasp of a community of researchers working together.

Moreover, genomic technology favors community research in a deeper way, as the amount of raw data produced by a single experiment often extends far beyond the interests and goals of the original experimenter. If the data are collected and archived in systematic fashion, it may be possible to extract new and unanticipated insight from formal meta-analyses that extend across multiple experiments. High-throughput microarray data in particular lends itself to systematic archiving, because of its structured quantitative nature.

Organizing a truly useful database of gene expression built from the efforts of multiple independent investigators requires an unusual level of formal planning and rigorous documentation of experimental details. This is especially critical for experiments involving animal behavior. As the now-classic results of Crabbe et al. (Science 284:1670; 1999) have shown, even when investigators in different laboratories think they are precisely replicating a common experimental protocol, subtle uncontrolled differences of approach, interpretation and detail can lead to significant and unexpected differences in outcome. Such differences have the potential to be informative, but only if they are adequately documented in a way that allows meaningful comparison of results obtained in different laboratories by different investigators.

The broad goal of the SoNG Initiative is to bring these perspectives and opportunities to the community of investigators studying songbird neurobiology. Songbird studies have had major impact on many areas of biological and biomedical research, including the study of adult neurogenesis, steroid effects on brain function, sex-specific neural pathway development, "critical period" learning, and experience-dependent gene activation in the CNS. More than 50 laboratories are currently engaged in songbird studies in the U.S. alone, with annual NIH support at the level of ~$10 million. We believe the best is yet to come, as the rich natural biology of oscines affords yet-untapped opportunities for discerning how evolution, social organization, environment, experience, and the genome interact to guide how animals behave and adapt.

However, if the songbird research community is to remain competitive for attracting resources in this post-genomic era, it is imperative that we keep up with the advance of science technology. The availability (or lack thereof) of modern genomic tools and resources will have a lasting impact on our ability to attract students, post-docs, faculty positions, research grants, and pages in the most prestigious and competitive science journals.

The Specific Opportunity (and Challenge) Before Us Now

Songbird researchers are coming relatively late to the enterprise of high throughput genomics. 48 species are already represented with Unigene sets on the NCBI server, and only one of them is even a bird (the chicken). In some ways this is an advantage, as we now have the benefit of experienced colleagues, reduced costs, and established infrastructure for developing our own genomics tools and experiments. But this also has another implication: we don't want to repeat the same mistakes other communities have made. And we need to be thinking about how we can raise the bar to attract the attention and gain the respect of hard-bitten genomics pros who now edit the journals and staff the sequencing centers and granting agencies that we ultimately depend upon.

In consulting with these pros, one message has consistently come through: **we need to organize our community so that we get the most out of our collective efforts.** This is what the NIH is looking for in regards to future funding, whether it be for a BAC sequencing project within one of the Genome Centers, or a simple renewal of the RO1 grant that the SoNG Initiative started with.

And with 1000 standardized microarrays suddenly available to us, we have a golden opportunity to do just this. The statistical power of a microarray experiment is directly related to the number of independent biological samples that can be compared in controlled fashion. (Note that we're NOT talking about replicate measurements of a single sample – from the many precedents in the microarray field, the critical unit of replication is the biological sample, not the physical microarray). With the large number of investigators in the songbird community, and an initial large set of microarrays to work with, we are in a unique position to organize our collective efforts so that every investigator's experiments contribute to a larger database of microarray data. Even if every piece of data in the larger database is initially collected as part of a small single-investigator project, a secondary meta-analysis of the database can generate a larger, multivariable description of gene expression in the songbird brain and how it varies along many dimensions (e.g., age, sex, season, brain region, cell type, species). We believe this is something our community could produce with the resources now on hand, within the next couple of years if we act intelligently. And we believe such a product would be of extraordinary scientific value, and of great interest to the scientific press. Laying the infrastructure for such a possibility does not necessarily diminish the individual investigator's autonomy with respect to publication or choice of individual research question – **but it does require that effort is spent at the very beginning to systematize the collection of data.** This is a major goal of the SoNG Initiative.

III. Core Members of the SoNG Initiative

The SoNG Initiative was officially inaugurated January 1, 2003, with the support of NIH grant NS045264. From its inception, the SoNG Initiative has been conceived as an **open collaboration across the community of scientists engaged in the study of songbirds.** Operationally, the SoNG Initiative is centered at the University of Illinois, which has made a major commitment to genomics research across a wide diversity of organisms, ranging from microbes and plants to honeybees and agricultural animals. This commitment is reflected in the foundation of the W.H. Keck Center for Comparative and Functional Genomics, and more recently in the organization of the new Institute for Genomic Biology, both under the direction of Prof. Harris Lewin, an advisor to the SoNG Initiative. The Keck Center established an efficient pipeline for production of EST databases and microarrays from diverse species, and this pipeline has been critical to the rapid development of the resources now available to the songbird community through the SoNG Initiative. More information about the SoNG Initiative, the Keck Center, and the Institute for Genomic Biology can be found on the internet:

SoNG Initiative home page: http://titan.biotec.uiuc.edu/songbird/
Keck Center: http://www.biotec.uiuc.edu/keck.shtml
Institute for Genomic Biology: http://www.igb.uiuc.edu/

The current core participants in the SoNG Initiative are listed below.

Songbird Researchers

David Clayton, University of Illinois (Project P.I.)
Kirstin Replogle, Coordinator
Art Arnold (UCLA)
Gregory Ball (Johns Hopkins)
Eliot Brenowitz (University of Washington)
Julia George (University of Illinois)
Claudio Mello (Oregon Health and Sciences University)
Juli Wade (Michigan State University)

Statistician

Sandra Rodriguez-Zas (University of Illinois, Depts. of Statistics and Animal Sciences)

Units of the Keck Center for Comparative and Functional Genomics (U. Illinois)

High-throughput DNA Sequencing (Ryan Kim, Director; Alvaro Hernandez, Assistant Director)

Functional Genomics (Mark Band, Director)

Bioinformatics (Lei Liu, Director)

Other Consultants and Advisors

Gene Robinson (University of Illinois, Director of Neuroscience Program)

Charlie Whitfield (University of Illinois, Dept. of Entomology)

Bruce Schatz (University of Illinois, School of Library and Information Science)

Harris Lewin (University of Illinois, Founding Director of the Keck Center, and currently Director of the Institute for Genomic Biology)

IV. Specific Resources Now Available

A. ESTIMA database of gene sequences expressed in zebra finch brain.

The database may be accessed directly via this URL:

http://titan.biotec.uiuc.edu/cgi-bin/ESTWebsite/estima_start?seqSet=songbird

The database currently contains data from high-throughput sequencing of >34,000 independent cDNA clones (single reads, from the 5' end of the insert). The cDNA libraries used for this were constructed from polyadenylated RNA pooled from zebra finches of both sexes, ranging in age from embryonic day 30 up to adulthood. Using standard clustering and "contig" algorithms, these ESTs appear to represent the products of ~18,000 non-redundant genes. These gene products have been annotated by BLAST sequence similarity searches against four external databases: TIGR Gallus gallus (chicken) EST, NCBI chicken unigene, Swissprot, NR.aa. Approximately 76% of these zebra finches ESTs have highly significant hits against the chicken EST collection. In a nucleotide:nucleotide alignment (BLASTN) against the full chicken genome database (http://www.ensembl.org/Gallus_gallus/) ~72% of the zebra finch ESTs have highly significant hits. More details about the libraries, ESTs and methodologies can be found at the database URL above.

The database is searchable via an online software interface developed by Lei Liu and colleagues at the Keck Center, called ESTIMA (EST Information Management and Annotation tool). Via ESTIMA, one can retrieve EST sequence files and annotations by sequence ID, direct BLAST search, Gene Ontology terms, or keywords (against description fields imported from the external databases during the annotation process).

Individual EST clones may be purchased at nominal cost from the Clemson University Genomics Institute.

B. MICROARRAY of genes expressed in songbird brain

Single EST clones representing each of the inferred 18,000 non-redundant genes in the EST collection were amplified by PCR (by Kirstin Replogle) and spotted onto glass slides (by Mark Band and colleagues at the Keck Center). Individual cloned cDNAs were also contributed independently for specific genes of established interest, including the androgen receptor,

estrogen receptors alpha and beta; retinoic acid receptors alpha, beta and gamma; and the set of glutamate receptor subunits recently described by Wada, Sakaguchi, Jarvis & Hagiwara (GluR1-7, mGluR1-5, 8; KA1, KA2, NR1, 2A-D, 3; J Comp Neurol 476:44-64). Additional control DNAs on each array include 3 negative-control soybean genes not present in the songbird genome, and redundant spottings of several positive-control songbird reference cDNAs. These microarrays are now being qualified for hybridization against several other oscine species. Results of these studies will be made available to the community informally within the next two months or so.

We anticipate printing a total of at least 1000 replicates of this microarray, by the end of 2004. We will make these arrays available to the research community as follows, according to all requests made to the SoNG Initiative by the deadline of January 15, 2005.

1. For Community Collaborations: we will reserve at least 600 arrays for use in Community Collaborations. Investigators may propose a Community Collaboration as described below. Costs of these microarrays will be borne in full by the SoNG Initiative.
2. For direct purchase: we will reserve at least 200 arrays for direct purchase, by investigators who do not wish to engage in a Community Collaboration. The unit cost for direct purchase will be $100 plus shipping charges.

If unrequested microarrays remain after the January 15, 2005 deadline, the core members of the SoNG Initiative will establish and make public new priorities for their use and distribution.

C. COMMUNITY OF COLLABORATORS: Materials, labor and technical expertise for microarray experiments.

A major goal of the SoNG Initiative is to promote efficient, collaborative interactions in the community of songbird researchers, as the best means to embrace and exploit the new and rapidly developing technologies of high-throughput genomics. Microarray resources will be deployed with this philosophy in mind. Individual investigators may propose experimental comparisons of songbirds using microarrays, even if they have limited experience and resources themselves for microarray experiments. Experimental designs may be refined through interactions with core members of the SoNG Initiative. Materials (including arrays and reagents for hybridization probe labeling), labor, and technical expertise for execution and interpretation of microarray hybridizations will be provided by the SoNG Initiative. In most cases hybridizations will be performed at the University of Illinois, by personnel in the Keck Center for Comparative and Functional Genomics and the laboratory of David Clayton.

D. SONGBIRD NEUROGENOMICS DATA ARCHIVE

Another major goal of the SoNG Initiative is to promote the rational, systematic organization of songbird microarray data. To that end, all microarray hybridization data will be stored and made available to experimenters (and eventually the public) via a web-accessible array hybridization database now under development. All users of SoNG Initiative microarrays will be expected to approve and respect two conditions (see Application Forms for specific details):

All microarray hybridization data must be deposited in the SoNG Initiative database, and made available to the public in timely fashion.

All microarray hybridization experiments must be **thoroughly** and **systematically** documented, following a detailed format specified by the SoNG Initiative.

NOTE: the SoNG Initiative documentation standard may be considerably more detailed and rigorous than many investigators are used to following, in the course of their own independent research. The rationale for this rigorous, formal documentation is presented in the Background section, above.

V. Application Process

As described in Section I, experimenters may obtain access to the microarray resources of the SoNG Initiative in either of two ways:

1) by proposing a community collaboration;
2) by direct purchase of microarrays.

All potential users of SoNG Initiative microarrays – **including core members of the SoNG Initiative itself** – must complete and submit an application, and the application must be reviewed and approved as described below before any microarray experiment will be initiated. Specific application forms for each mechanism are appended.

Completed applications should be sent as an email attachment to: dclayton@uiuc.edu An acknowledgment of receipt will be sent by return email.

Deadlines: to receive full and equal consideration, an application must be received by **January 15, 2005.** Priorities for resource usage will be established based on the applications received by that deadline.

VI. Review process

All applications will be reviewed by the core members of the SoNG Initiative (Section III). Individual members of the SoNG Initiative must also submit application proposing specific experiments, and these will be reviewed by the group as a whole in the same manner as will all other applications. Criteria for ranking proposals and establishing priorities for resource utilization are as follows.

Review Criteria

1. **Potential impact**
   - perceived significance of the problem under study.
   - relevance and utility of microarray approach to the problem.
   - soundness of proposed experimental design.
2. **Efficiency of Resource Utilization**
   - “bang for the buck” – how expensive will the experiment be, relative to the potential reward?
   - Complementarity and synergy with other proposed experiments.
3. **Overall Programmatic balance**

   Below we outline five broad questions that characterize the current study of songbird neurogenomics. A priority is to encourage the development of experiments that collectively address all of these:

   - Comparative: what genomic changes underlie the different patterns of song production, plasticity, song circuit organization and related social behavior observed in different passerine species?

- Brain Sex, Gender and Hormones: what genes, genomic pathways and hormonal signals underlie the "male" versus "female" patterns of song circuit formation?
- Critical Period Learning: what is configurationally different about the song system (and the genes expressed) during the different phases of juvenile song learning and adult crystallization?
- Activational: what is the function of gene activation during adult listening and singing, and how might gene activation provide insight into functional organization of the nervous system?
- Environmental: what is configurationally different about the song system (and the genes expressed) when birds are exposed to different environmental contexts (day length, humidity, social structures)?

Application: Proposal for a Community Collaboration

Please follow the format below and complete all items. You can cut and paste the application form text as a template. You may take as much space as you need to answer each question.

A. Proposer's Contact Information

Proposer's Name:
Institution:
Email Address:
Telephone:
Date of Submission:
Other Co-investigators Involved in Biological Aspects of Experimental Design & Execution:

B. Tell us now:

- Question asked and/or hypothesis to be tested
- Species
- Gender(s)
- Age(s)
- Dissected Brain Region(s)
- Method for verifying accuracy of dissection
- Anticipated weight or volume of each dissected sample
- Number of experimental groups ("main effects")
- Number of biological replicates (animals) per group
- Rationale or any preliminary data for gene regulation

C. Certify that you will tell us later:

Please indicate (YES or NO) whether you accept and agree with the following statement: "By submitting this application, I (the Proposer) certify that I have read, understand and accept the rationale provided for detailed documentation of microarray experiments, as presented in the Call for Proposals. I will provide the following information to the Collaborators, describing the tissues as I provide them for microarray analysis."

For each Bird:

- Source (birthplace and supplier)
- Age (including birthdate if known)
- Gender
- A digital color photograph (capturing coloration and any distinctive physical features).
- Light cycle (Day:Night ratio in the week prior to sacrifice)
- Recent social conditions

  - open aviary or bird cage?
  - How many other birds, and of what genders, in the cage and/or room?
- Housing location relative to outside (e.g., underground, above ground with windows, windowless, drafty, indoor air)
- Outside weather at time of sacrifice (temperature, humidity or precipitation)
- Health at time of sacrifice
- Any unusual behavioral character at time of sacrifice (e.g., calm? Agitated?)
- Time of Day of Sacrifice
- Describe the Place of Sacrifice
  - Size of room,
  - Light intensity
  - Light quality (i.e., natural, fluorescent, incandescent, qualitative brightness)
  - Sound quality
  - background noise (white-noise vs biologically agitating noise)
- Precise method of euthanasia
- Housing, Dietary, Environmental and Social History
    - Overview from birth
    - Detailed record of experience during the week prior to sacrifice
- Who did the sacrifice, and how cleanly, and how quickly
- Time between death and tissue collection
- Who did the dissection
- Dissections: name of region(s), approx volume or weight of each piece of tissue
- Intended Experimental Manipulation (group)

D. Certify Intent to Share Data

Please indicate (YES or NO) whether you accept and agree with the following statement: "By submitting this application, I (the Proposer) certify that I understand and will abide by the following conditions for storing and sharing data from these experiments. "

- Complete documentation of experimental design, biological sources, microarray hybridization conditions, and resulting data (original .GPR files) will be collected by (or immediately provided to) the Songbird Neurogenomics Data Archive.
- Access to these data will be limited to the Proposer and the Collaborators until first publication of data resulting from these hybridizations.
- Upon first publication, all data from these hybridizations will enter the public domain via Songbird Neurogenomics Data Archive and/or deposition in other publically maintained microarray databases.

E. Certify the Understanding of Anticipated Authorship

Please indicate (YES or NO) whether you accept and agree with the following statement:

“By submitting this application, I (the Proposer) certify that I understand and will abide by the following conditions for authorship of any publications resulting from this work. “

The Proposer retains the right to direct the first publication of data resulting from these hybridizations (subject to the specific exclusion below*).

Collaborating members of the SoNG Initiative retain rights to authorship on the first publication. Authorship will be restricted to the specific individual(s) in the SoNG Initiative who actually performed a direct and significant role in planning, execution and analysis of the experiments.

The Proposer retains the right to determine order of authorship on the first paper.

After the first publication, the data enter the public domain and may be used independently by the Proposer, the Collaborators, or anyone else (subject to the usual conventions of resource acknowledgement and referencing).

*SoNG Initiative retains the right to direct publication of a meta-analysis of the combined data in the complete set of all experiments arising from the current microarray release. In the event the Proposer has not published the results of his/her specific study within 6 months of the time all microarrays in the current release have been hybridized and scanned and entered in the Data Archive, the Collaborators will notify the Proposer of their intent to publish the meta-analysis. The Proposer will be given the option of co-authorship on the meta-analysis publication (to be directed by the Collaborators). Publication of the meta-analysis will then constitute “first publication” and the data will enter the public domain.

Application for Microarray Purchase

A. Proposer's Contact Information

Proposer's Name:

Institution:

Email Address:

Telephone:

Date of Submission:

B. Number of Arrays Desired:

C. Certify Intent to Share Data

Please indicate (YES or NO) whether you accept and agree with the following statement: "By submitting this application, I (the Proposer) certify that I understand and will abide by the following conditions for storing and sharing data from these experiments. "

At the time of publication of data resulting from use of these microarrays, or within 1 year of data generation, the Purchaser agrees to provide a) original .GPR files, and b) complete documentation of experimental design, biological sources, and microarray hybridization conditions, to the Songbird Neurogenomics Data Archive, where it will be maintained in the public domain.

D. Certify Willingness and Ability to provide documentation

Please indicate (YES or NO) whether you accept and agree with the following statement: "By submitting this application, I (the Proposer) certify that I have read, understand and accept the rationale provided for detailed documentation of microarray experiments, as presented in the Call for Proposals. I will provide the following information to the Collaborators, describing the tissues as I provide them for microarray analysis and the conditions of each microarray hybridization."

For each Bird:

- Source (birthplace and supplier)
- Age (including birthdate if known)
- Gender
- A digital color photograph (capturing coloration and any distinctive physical features).

- Light cycle (Day:Night ratio in the week prior to sacrifice)
- Recent social conditions
  - open aviary or bird cage?
  - How many other birds, and of what genders, in the cage and/or room?
- Housing location relative to outside (e.g., underground, above ground with windows, windowless, drafty, indoor air)
- Outside weather at time of sacrifice (temperature, humidity or precipitation)
- Health at time of sacrifice
- Any unusual behavioral character at time of sacrifice (e.g., calm? Agitated?)
- Time of Day of Sacrifice
- Describe the Place of Sacrifice
  - Size of room,
  - Light intensity
  - Light quality (i.e., natural, fluorescent, incandescent, qualitative brightness)
  - Sound quality
  - background noise (white-noise vs biologically agitating noise)
- Precise method of euthanasia
- Housing, Dietary, Environmental and Social History
    - Overview from birth
    - Detailed record of experience during the week prior to sacrifice
- Who did the sacrifice, and how cleanly, and how quickly
- Time between death and tissue collection
- Who did the dissection
- Dissections:
  - name of region(s)
  - approx volume or weight of each piece of tissue
  - method of verification of accuracy of dissection
- Intended Experimental Manipulation (group)

For each Slide:

- Date of hybridization
- Identification of both probes used
- Which probe is labeled with which dye?
- Protocol for nucleic acid extraction
- Protocol for probe labeling
- Protocol for microarray hybridization
- Protocol for microarray data collection (scanning)
- Copy of original .GPR file

FAQs (Frequently Asked Questions)

The SoNG Initiative is designed as a collaborative, community enterprise. Participating investigators bring many different perspectives and agendas to the project. The questions and responses below reflect issues that have been raised in discussions among members of the SoNG core group. Many of these issues are still evolving. Please feel free to continue to raise questions!

Resources and Availability

Let me get this straight – if I participate in a community collaboration, your SoNG grant pays for everything after tissue dissection – not only the costs of the microarrays but also the RNA extraction, probe labeling, hybridization and data analysis? And you do all that work for me?

Yes, precisely.

What does it cost to perform a microarray hybridization?

In terms of Direct Costs charged to the SoNG grant, the cost of producing one array in this project is approximately $100 (including proportionate cost-sharing for primary laboratory personnel at the bench). This does not include the expense of producing the original cDNA libraries and EST sequence database.

Each "2-color" hybridization will involve producing labeled probes from two RNA samples – for example, one experimental sample and a common Reference against which all data are compared. Current cost for labeling the two probes for a single microarray hybridization, plus the costs of other hybridization reagents and array scanning, is approximately $100.

Thus obtaining a standardized measurement of gene expression from a single brain region of a single animal (against a common reference) costs approximately $200.

Current experience indicates that it is important to obtain multiple independent biological replications for each experimental group. On the other hand, it is less important to obtain multiple independent technical replications (i.e., multiple hybridizations of the same RNA sample). Hence, for a single 2-group comparison using 6 separate animals for each group (biological n=6 per group), measured against a single common Reference sample, 12 arrays would be required, at a total experimental cost of approximately $2400.

Can you really afford to pay for all this?

We certainly have enough funds to complete many microarray experiments, based on the initial NIH award.

What if there are more good experiments than arrays?

We already suspect this will be so. In our initial surveys, more than 30 different investigators expressed an interest in using our arrays, and we project a potential immediate demand for as many as 1500-2000 arrays. That is well beyond what we were funded to produce in this initial 3 year period.

Major motivations of our current organizational approach are: 1) to establish a rational and fair mechanism for distributing the arrays as widely as possible, 2) maximize the quality and impact of the resulting data, 3) lay the groundwork to attract more funds into this arena, to continue and extend the research.

How many separate experiments does your group expect to support with these arrays?

Considering n=6 biological replicates per treatment group in a reference design and an average of 3-4 groups per experiment, we expect to proceed with a total of about 25-35 experiments with the current set of 600 arrays.

Can we raise more money to keep this going?

We are planning to submit a competing renewal in March 2005, and that renewal will take into account any unmet projected budgetary needs for completing all the experiments that have been formally proposed by the community in response to this Call.

The overall objectives are fine, but my own interest is really much more narrow, and I'm not even especially interested in most of the 18,000 genes on the array – only a subset. How can you help me?

You can make your own sub-array on your own, using selected sequences from our ESTIMA collection. You would bear the cost of producing such an array, but we can provide advice on how to approach this, if desired.

Experimental Design

How many animals do I need for a single experimental group?

Assuming you are making measurements in a small well-defined brain region (e.g., a song nucleus), and you want to obtain some measure of biological variability, and you are willing to see your RNA samples amplified prior to hybridization (see below), then you are in a position to proceed as for other sorts of experiments. We like to shoot for a minimum biological n=6 per group, although robust effects may begin to emerge at smaller sample sizes.

For each group, should I pool my samples, or run a separate array for each one?

All else being equal, there is an advantage to running them as separate samples on different arrays. The advantage is that you get true biological replication, which gives you a measure not only of the mean but also the variance for each gene. Now, having the variance may not end up being important to you. But in some microarray studies it turns out to be informative. In some cases people have observed that the genes that show the greatest expression differences "on average" typically turn out to have very high variances -- they're way up in a few samples, and maybe even down in some others. Stress response genes in particular may show this pattern, especially from samples collected from the field. Their variability may be due to uncontrolled environmental factors, like temperature and weather and nutritional status on the day of collection. Unless you are specifically interested in studying stress responses, it may be more informative to you to focus on genes that are differentially expressed at lower ratios -- but are CONSISTENT across the whole population. The point is that if you pool your samples, you can't extract this kind of information.

A countervailing consideration applies have you have a large number of groups you want to compare. Suppose you'd really like to look at 3 different song nuclei, separated into 2 sexes, and monitored at 5 timepoints. That would be 3 x 2 x 5 = 30 groups. With our recommended minimum group n=6, that would burn up 180 arrays – nearly a third of our current working budget of 600 arrays! In that case you might be better off pooling samples for each timepoint. This way you'd be able to get a measure on all 30 groups, and the pooling would minimize the impact of random variability (though it will also eliminate your ability to measure the variability).

For a community collaboration, what would I provide you with – tissue, or RNA?

We would prefer you send us dissected tissue, appropriately preserved (i.e., stored in RNAlater). We will make the RNA using a standardized protocol to minimize sample-to-sample variability.

How much tissue (or RNA) do I need to obtain for each microarray hybridization?

Our current standard conservative protocol uses 10 micrograms of total RNA to produce one labeled probe for a single microarray. (Note that a second probe will also be applied to the same slide, labeled with a different fluorescent color; in most cases this will be derived from a single common Reference RNA sample to be used across all experiments supported by the SoNG Initiative).

As a rule of thumb, a typical song nucleus is about 1 microliter in volume and produces about 1 microgram of total RNA. Hence, for experimental analysis of individual song nuclei, one of two strategies is required: 1) pooling samples; 2) probe amplification

If birds are not in limiting supply, it is certainly feasible to pool samples. However, according to our experience above, it may take song nuclei from 10 animals to obtain enough RNA for a single microarray. A single such hybridization allows no technical replication of the hybridization itself, nor does it provide any measure of biological variability, which is often important in the final statistical analysis and interpretation. Thus, to obtain a robust measure of the experimental condition of interest, tens or even hundreds of birds might be required, assuming very precise dissections of a single nucleus are to be used.

The alternative – and in our experience it is strongly favored – is to employ procedures that are now fairly standard for amplifying the RNA obtained from small samples (e.g., Agilent's kit; Whitfield et al., Science 302:296; 2003). **The RNA from a single song nucleus can be amplified to provide enough probe for 1-3 independent microarray hybridizations.** The downside is that amplification inevitably risks the introduction of some artifacts or data compression. However, in our experience to date, this risk is relatively small compared to the impracticalities of obtaining, e.g., dissected samples from 100 zebra finches for each experimental group.

These methods are still evolving, and we will continue to communicate with the community as things (hopefully) continue to improve.

What's the best way to verify brain region dissections?

We request that investigators verify their dissections by Nissl-staining of the remaining tissue where possible, and provide those results as part of the documentation process.

In my proposal, do I need to consider looped versus reference strategies for experimental design?

Not necessarily. Because of the distributed nature of this research program, and the interest in comparing samples obtained in different laboratories and experiments, our intention is to develop a single universal Reference standard against which most or all experimental samples (and certainly all experiments) will be compared. The Reference standard is simply a large batch of RNA from a single, defined biological condition. In a standard microarray experiment, each array is hybridized against two cDNA preparations labeled with complementary fluorophores. One cDNA is derived from one experimental sample, and the other cDNA is derived from the universal Reference RNA. The Reference RNA is used to provide a consistent normalized standard of reference for comparing the intensities of hybridization signals across different microarray slides. We may consider looped (or daisy

chain) design in some cases, but we would still want to obtain data against the Reference standard to allow bridging to the rest of the data in the Data Archive. The precise details of each microarray hybridization experimental design will be worked out once the aggregate set of experiments has been established in February 2005.

I just want to compare two specific groups. For my immediate purpose, wouldn't it be better just to do a direct comparison of samples from the two groups on each microarray, instead of indirectly comparing them via an arbitrary reference?

There are a number of different designs for microarray experiments, and the appropriateness of each depends on the specifics of the experimental situation (e.g., number of experimental units and observations; number and type of factors, covariates and interactions to test; desired power; biological sample variability; amount of time and resources available for the project). A global treatment of the various alternatives is well beyond the scope of this FAQ. Our focus here is on optimizing the deployment of resources for this particular project – and as described in the Background section, a major goal the SoNG Initiative is to organize our experiments in such a way that we can create a mega-dataset that combines everyone's microarray work. This goal leads us inevitably to favor designs that are built around a universal reference. We recognize that there may be some specific experimental circumstances that warrant use of some direct comparisons; in those cases we would suggest a mixed design where at least some of the arrays are hybridized to the reference standard to allow connection of the local experimental data to the larger mega-dataset.

What's the reference sample going to be?

At this writing, we favor the idea of using whole brain RNA from non-breeding female zebra finches from a single colony, isolated in single cages. Two important points to appreciate about the reference sample: 1) In many respects, its identity is arbitrary – it can be regarded simply as a consistent internal standard used for normalizing data collected on different microarray slides. 2) Even if we create a large quantity of the reference standard at the start, it is important to anticipate the potential need to regenerate the reference standard as accurately as possible again in the future, if needed (e.g., the reference is lost or degraded or used up). For that reason it is desirable to have a reference that is as homogenous and uniform as possible.

What about hybridizations against species other than zebra finch – do you still prefer use of the zebra finch Universal Reference?

Yes, for two reasons. First, if the cross-hybridizing species gives a much weaker signal than the zebra finch reference against a particular gene, this may indicate that the sequences are too divergent to be reliably measured by cross-hybridization; this is an important "quality control" point to keep in mind in interpreting the data. Experiments are currently underway to assess the fidelity of cross-hybridization against other avian species using genomic DNA as the labeled probe (to avoid the confound of possible species-specific differences in expression level), and we will report on our findings as soon as we have them. Second, remember again that the choice of reference is somewhat arbitrary. Direct hybridization against the zebra finch reference will connect the other species' data in a way that may allow investigations of possible species differences in gene expression and conservation.

Where can I get more information about microarray experimental designs?

Here's a brief, general introduction:

- Churchill, G.A., 2002, Fundamentals of experimental design for cDNA microarrays. Nature Genetics 32(Suppl.), 490-495.

These deal more specifically with issues related to reference-based designs:

- Puskas LG, Zvara A, Hackler L Jr, Micsik T, van Hummelen P (2002). Production of bulk amounts of universal RNA for DNA microarrays. Biotechniques. 2002 Oct;33(4):898-900, 902, 904.
- Yang IV, et al. (2002). Within the fold: assessing differential expression measures and reproducibility in microarray assays. Genome Biology, 3(11):R0062.1-0062.12.
- Park PJ, et al. (2004). Current issues for DNA microarrays: platform comparison, double linear amplification, and universal RNA reference. Journal of Biotechnology 112: 225-245.
- He X-R, Zhang C, Patterson C (2004) Universal mouse reference RNA derived from neonatal mice. BioTechniques 37:464

Review Process

Who will carry out the review?

The core members of the SoNG Initiative will carry out the review process, as a committee. David Clayton will function as Chair of the review committee.

Who will review the proposals that originate from members of the review committee?

The review committee will be responsible for considering their own proposed experiments on an equal footing with those of other investigators not on the committee. We recognize that this may seem at first glance to pose a conflict of interest. However, the Review Committee comprises the investigators entrusted by the NIH with responsibility for this project. Note that investigators have the option of purchasing arrays directly, without participating in the collaborative review process. Beyond that, we are offering to share ALL of our resources project with the community at large, in an organized fashion, out of the conviction that this will have the most beneficial impact on our field as a whole and on our own individual research programs.

What kind of feedback can I expect to receive on my proposal?

We will establish a rank order, and make that information generally available.

The ranking will be based on the criteria set out under Section VI, and will not be subject to appeal during the next calendar year.

We may offer specific suggestions about ways to improve an experiment, or may suggest some sort of recombining of efforts where it is clear that two different groups want to do related experiments.

What if 10 different labs all propose a similar experiment – how will you decide who is the formal "proposer" in the context of future publication?

If the SoNG Initiative decides to offer microarray resources to any investigator, that investigator has the right to independent primary publication of the data generated from his or her samples, as stated in the application certifications. The investigator of course may also choose to co-publish with other collaborators, if it appears that this would result in a stronger or

more complete primary publication. (In this circumstance, authorship priorities would need to be negotiated on a case-by-case basis).

If multiple investigators propose essentially the same experiment, it may not be possible to support everyone with our current resources. In this case, the Review Committee will have to make decisions about who gets to use the arrays and who doesn't. We will attempt to distribute array usage as widely and as fairly as possible.

The broader objectives of the overall project should open up opportunities for multiple parallel investigations (by multiple investigators) all sharing a common immediate focus, by suggesting ways in which the investigations might be structured to support the broader meta-analysis. For example, some of the experimental variables we would like to assess in the long run are the reproducibility of experimental results in different labs, and the effects of environmental variables that are typically "controlled" but not necessarily analyzed directly in a single lab (e.g., source of birds; calendar or seasonal effects on zebra finches). Therefore we expect to allow some parallel, apparently redundant investigations to generate data that will support these secondary analyses.

What experiments are the individual members of the Review Committee already intending to propose?

The primary interests of the review committee members, in the context of the present microarray initiative, are listed below.

- Arnold: sex chromosome-linked gene expression in zebra finches
- Ball: anatomical, seasonal and comparative factors in steroid hormone-associated gene regulation
- Brenowitz: gene regulation associated with seasonal plasticity in species other than zebra finch
- Clayton: phases of gene activation following novel song playback in zebra finches
- George: synaptic plasticity gene regulation in the developing song circuit
- Mello: fine anatomical dissection of gene expression
- Wade: sexually dimorphic gene expression during zebra finch development
